# Spinochrome Identification and Quantification in Pacific Sea Urchin Shells, Coelomic Fluid and Eggs Using HPLC-DAD-MS

**DOI:** 10.3390/md19010021

**Published:** 2021-01-06

**Authors:** Elena A. Vasileva, Natalia P. Mishchenko, Van T. T. Tran, Hieu M. N. Vo, Sergey A. Fedoreyev

**Affiliations:** 1 Laboratory of the Chemistry of Natural Quinonoid Compounds, G.B. Elyakov Pacific Institute of Bioorganic Chemistry, Far Eastern Branch of Russian Academy of Sciences, 690022 Vladivostok, Russia; mischenkonp@mail.ru (N.P.M.); fedoreev-s@mail.ru (S.A.F.); 2 Nhatrang Institute of Technology Research and Application, VAST, Khanh Hoa 650000, Vietnam; vanvvlnt@yahoo.com.vn (V.T.T.T.); nhuhieu@nitra.vast.vn (H.M.N.V.)

**Keywords:** sea urchins, quinonoid pigments, spinochromes, HPLC-DAD-MS, quantification, coelomic fluid, eggs, embryos

## Abstract

The high-performance liquid chromatography method coupled with diode array and mass spectrometric detector (HPLC-DAD-MS) method for quinonoid pigment identification and quantification in sea urchin samples was developed and validated. The composition and quantitative ratio of the quinonoid pigments of the shells of 16 species of sea urchins, collected in the temperate (Sea of Japan) and tropical (South-China Sea) climatic zones of the Pacific Ocean over several years, were studied. The compositions of the quinonoid pigments of sea urchins *Maretia planulata*, *Scaphechinus griseus*, *Laganum decagonale* and *Phyllacanthus imperialis* were studied for the first time. A study of the composition of the quinonoid pigments of the coelomic fluid of ten species of sea urchins was conducted. The composition of quinonoid pigments of *Echinarachnius parma* jelly-like egg membrane, of *Scaphechinus mirabilis* developing embryos and pluteus, was reported for the first time. In the case of *Scaphechinus mirabilis*, we have shown that the compositions of pigment granules of the shell epidermis, coelomic fluid, egg membrane, developing embryos and pluteus are different, which should enable a fuller understanding of the functions of pigments at different stages of life.

## 1. Introduction

The red granules discovered by MacMunn in the perivisceral fluid of the sea urchin *Echinus esculentus* over a century ago inspired research on the composition, structure, function and biological activity of the quinonoid pigments they contained, and still attract the attention of chemists and biologists [1]. Today, it is known that pigment granules are mostly concentrated in the epidermis, covering the shell and needles of the sea urchin, and its pigment pattern is characteristic of the species [2,3]. Pigment granules have been found in red spherule cells in the coelomic fluid (CF) for all species of sea urchins. In healthy specimens, the rest of the CF is not colored. For a long time, it was assumed that echinochrome A was the main pigment in the composition of red spherule cells, and plays a protective role against bacteria [4]. Thus, during bacterial or viral infection, as well as during injury and other damage to the organs of the sea urchin, pigments are released from red spherulocytes and the encapsulation of foreign objects occurs. Pigment granules were found in the jelly-like membrane covering the eggs of many, but not all, species of sea urchins. The number of granules and their location in the egg membrane (at the edge or in the centre) can also be a species characteristic. Thus, sea urchins of the order Clypeasteroida (*Scaphechinus griseus*, *Scaphechinus mirabilis*, and *Echinarachnius parma*) have from 15 to 40 pigment-containing chromatophores in the jelly-like egg membrane, the largest number being found in *S. mirabilis* eggs [5]. The brightly colored granules are easily detectable under an optical microscope in eggs, CF, gastrulating embryos and the larvae of *S. mirabilis* [6,7].

Spinochromes, the quinonoid pigments of sea urchins, are known for their diverse pharmacological effects: antioxidant [8,9,10,11], antimicrobial [12,13], antiviral [14], cardioprotective [15], neuroprotective, among others [16,17,18]. Despite numerous biochemical studies of spinochromes and examples of their use in medicinal practice, the physiological functions of these compounds in sea urchins are far from understood. The reason for this is the lack of sufficient information on the qualitative and quantitative composition of spinochromes in the body of sea urchins at different life stages, and on the influence of environmental factors on their content. At present, the capabilities of various mass spectrometric analytical techniques make it possible to study the composition and quantitative content of compounds, with high sensitivity for small amounts of natural samples. This work is the result of many years of research on quinonoid pigments of 16 species of sea urchins, collected in various regions of the Pacific Ocean, using the validated high performance liquid chromatography method coupled with diode array and mass spectrometric detector (HPLC-DAD-MS) method. We also present an attempt to analyze the structural diversity of spinochromes described in the literature.

## 2. Results and Discussion

### 2.1. Spinochrome Isolation

In this work, aqueous ethanol (70%) containing sulfuric acid (10%) was used to extract pigments from the shells of sea urchins. From the crude extracts concentrated under reduced pressure, quinonoid pigments were sequentially extracted, first with chloroform and then ethyl acetate. This extraction was used in order to separate the sum of pigments into two fractions, each containing groups of pigments with different polarities, and to provide a fuller extraction of minor quinonoid pigments. Chloroform and ethyl acetate extracts were combined and analyzed, to determine the composition and quantitative content of spinochromes in the samples.

### 2.2. HPLC-DAD-MS Method Development and Validation

To identify quinonoid pigments and evaluate their content in sea urchins extracts, HPLC-DAD-MS was used. In the HPLC-DAD-MS method, we have developed HPLC conditions to successfully separate more than 20 quinonoid pigments of sea urchins during one short analysis. For its development, we used standard samples of spinochromes, the structures of which were previously established by us using NMR spectroscopy and high-resolution mass spectrometry [10,19,20,21,22,23]. The use of a diode array detector (DAD) makes it possible to obtain absorption spectra of spinochromes in the wavelength range from 200 to 700 nm, with characteristic absorption maxima for each compound. A mass spectrometric detector with electrospray ionization (ESI-MS) and a single quadrupole mass analyser was used, allowing [M + H]^+^ and [M − H]^–^ ion signals to be obtained. Validation of the HPLC method was performed, during which the following parameters were checked: specificity, linearity, limit of detection (LOD), and limit of quantification (LOQ) for standard samples, as well as accuracy and reproducibility of the method (Table 1 and Table S1).

The detection wavelength (λ) of 254 nm was selected based on all target compounds having intense absorption within the range λ = 235–280 nm. The high specificity and stability of the method is confirmed by extensive statistical analysis of real sample extracts from over 20 species of sea urchin. Due to preliminary preparation of the samples, the peaks of the target compounds did not overlap with the impurity peaks, and a sufficient pH level of the eluting system and column temperature control (40 °C) made it possible to obtain stable retention times of spinochromes with the columns used.

The linearity of the method was confirmed by the regression equations presented in Table 1 and by the correlation coefficients (R^2^), the values of which were greater than 0.99. The analytical region for echinochrome A (**16**) was defined as the range of 72–600 ng/mL, spinochrome D (**3**) within 134–800 ng/mL, spinochrome E (**1**) within 160–760 ng/mL, and 7,7-′anhydroethylidene-6,6′-bis(2,3,7-trihydroxynaphthazarin) (**11**) within 240–1500 ng/mL. The accuracy and reproducibility of the method for the quantitative determination of standard samples **1**, **3**, **11** and **16** are confirmed by the results shown in Table S1 (Supplementary Material).

Therefore, the analysis of chromatograms allows the expression of the relative content of each pigment in the extract as a molar percentage of the total pigment composition. To calculate the content of each compound, the peak area was used as the main parameter. 

**Figure 1:**
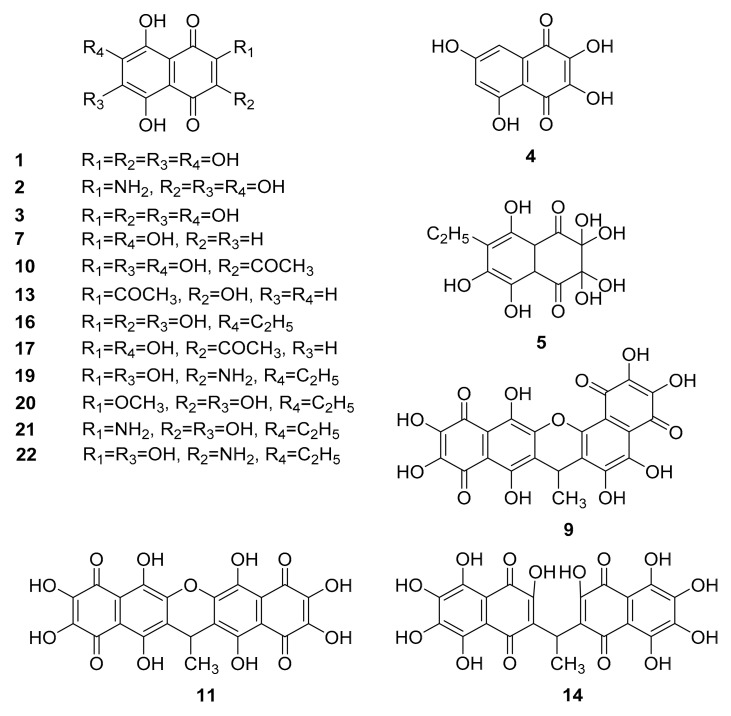
Structures of quinonoid pigments of sea urchins.

In our laboratory, the library of HPLC-MS parameters of spinochromes and their synthetic analogues was created and constantly updated. Identification of quinonoid pigments was performed on the basis of retention time, absorption spectra and [M − H]–ions *m/z* values, in comparison with data obtained from standard samples. HPLC-MS parameters of all compounds detected during this work are shown in Table 2, and the structures of known pigments are shown in Figure 1.

### 2.3. Quinonoid Pigments of Sea Urchin Shells and Spines

When discussing the contents of individual spinochromes in extracts of sea urchins, integer average values are given in the text, and statistically processed values are given in Table 3.

#### 2.3.1. Order Camarodonta

We have studied the composition of quinonoid pigments of representative sea urchins from two families of the Camarodonta order—Strongylocentrotidae and Toxopneustidae. From the Strongylocentrotidae family, the composition of pigments was determined for sea urchins from a temperate climatic zone, *Mesocentrotus nudus* and *Strongylocentrotus intermedius*. The Toxopneustidae family in this work was represented by two tropical species, namely *Toxopneustes pileolus* and *Tripneustes gratilla*.

##### Strongylocentrotidae

Sea urchins of the species *Mesocentrotus nudus* belong to the Strongylocentrotidae family, and are a dominant sea urchin species in the northwest Pacific. The main component of the total extract of *M. nudus* from the Sea of Japan was spinochrome E (**1**) (up to 54%), echinochrome A (**16**) (up to 18%), spinochromes A (**17**) (8.5%), B (**4**) (1%), C (**10**) (4%), D (**3**) (3%), and spinamine E (**2**) (approximately 3%), along with unknown pigment **18** (up to 8%) (Table 3). In the mass spectrum of this compound, a peak [M − H]^–^ of *m/z* 527 can be seen, and its absorption spectrum was distinguished by the presence of an additional absorption band in the long-wavelength region, at 542 nm (Table 2). The retention time, absorption and mass spectra of pigment **18** coincided with those of the synthetic sample of 6,7,8,9-tetrahydroxy-4-methyl-2-(3,5,6,7,8-pentahydroxy-1,4-dioxo-1,4-dihydronaphthalen-2-yl)-3,4-dihydro-2*H*-benzo[*g*]chromene-5,10-dione [24].

Previously, 3-acetyl-2,7-dihydroxy-6-methylnaphthazarin was isolated from the test and spines of *M. nudus* [25]. In addition, acetylaminotrihydroxynaphthoquinone was detected in *M. nudus* by using ultra-performance liquid chromatography (UPLC) [26].

Sea urchins of the species *Strongylocentrotus intermedius* are widespread in the Sea of Japan and in the southern part of the Sea of Okhotsk, at shallow depths (up to 25 m) [27]. In the total extract of *S. intermedius* from the Sea of Japan, the main pigment was binaphthoquinone **11** (up to 40%), and spinochromes D (**3**) (up to 19%), E (1) (10%), A (**17**) (about 1%), and binaphthoquinones **14** (7%) and **9** (6%) were also identified (Table 3, Figure S1). In addition to the known compounds, the extract contained compound **18** (up to 7%), same as in *M. nudus*. Previously, spinochromes A, B, C and ethylidene-6,6′-bis(2,3,7-trihydroxynaphthazarin) were isolated from this species of sea urchin, in samples also collected from the Sea of Japan [28]. Li et al., using macroporous resin extraction, isolated only spinochrome B from *S. intermedius* crude pigment extract [29]; however, this may be due to a low pigment desorption rate.

##### Toxopneustidae

Tropical sea urchins of the species *Tripneustes gratilla* and *Toxopneustes pileolus* have similar habitats in the seas of the Indo-Pacific, the Red Sea, near the Bahamas, and in Hawaii [30,31]. Both are “collector urchins”—they cover themselves with fragments of shells, corals and pieces of algae. 

The total obtained extract of *T. pileolus* from the South China Sea contained echinochrome A (**16**) (80%) as the main component, as well as spinochromes B (**4**), C (**10**), D (**3**) and binaphthoquinone **14**, in amounts of 4–6% (Table 3, Figure S2). Earlier, Koltsova et al. investigated the butanol extract of *T. pileolus* from the South China Sea [32], which was found to contain spinochromes A, B and C, and their content was dependent on the colour of the sea urchin: spinochrome B prevailed in green-brown individuals, and spinochrome A in bluish individuals. Brasseur et al. investigated the composition of pigments in the ether extract of *T. pileolus* collected near Madagascar, the main pigment was spinochrome B, while the minor components were spinochrome A-Iso 2, spinochrome D-Iso 1, spinochromes C and E, and echinochrome A [12].

In the chloroform extract of *T. gratilla* collected from the South China Sea, the main compound was spinochrome A (**17**) and small amounts of mompain (**7**) were also present, consistent with published data [3] (Figure S3). In the ethyl acetate extract of *T. gratilla*, spinochrome E (**1**) was first identified, and then its aminated analogue **2** (Figure S4). In the total extract, the pigment content was as follows: spinochrome E (**1**) (up to 67%), spinochrome A (**17**) (up to 26%), spinamine E (**2**) (up to 5%) and mompain (**7**) (approximately 2%) (Table 3). Brasseur et al., in the ether extract of *T. gratilla* collected near Madagascar, reported the presence of spinochrome D-Iso 3, spinochrome E, and echinochrome A [12].

#### 2.3.2. Order Cidaroida

The quinonoid pigments of the tropical sea urchin *Phyllacanthus imperialis* were studied here for the first time. According to HPLC-MS analysis, the *P. imperialis* ethyl acetate extract did not contain quinonoid pigments. Spinochromes A (**17**) (77%) and C (**10**) (23%) were present as the main compounds in the chloroform extract of *P. imperialis* (Table 3, Figure S5), as well as trace amounts of acetylaminotrihydroxynaphthoquinone **8** (up to 0.2%), previously detected by Zhou et al. in *S. nudus* [26].

#### 2.3.3. Order Diadematoida

##### Diadema Genus

*Diadema savignyi* and *D. setosum* are sympatric species of sea urchins with reproductive isolation; that is, they originate from the same population and occupy the same habitat, but have different breeding periods [33]. Due to their common origin, these species have a highly similar pigment composition.

The total extract of *D. savignyi* contained mainly echinochrome A (**16**) (83%), as well as seven minor pigments, five of which were identified as spinochromes E (**1**) (4%) and D (**3**) (2%), binaphthoquinone **11** (3%) and two echinochrome A monomethyl ethers **20** and **22** (approximately 1.5% each) (Table 3, Figure S6). In addition to the known pigments, an oxidation product of echinochrome A dehydroechinochrome (**5**) was discovered (approximately 3%). Pigment **12** (up to 1%) was also detected, with a typical naphthazarin spectrum and *m/z* [M − H]^–^ of 499, likely a spinochrome dimer (Table 2).

In the *D. setosum* extract, as in *D. savignyi*, the main pigment was echinochrome A (**16**) (92%), and spinochrome E (**1**) (up to 4%) and echinochrome A methyl ether **20** (up to 4%) were also present in small quantities (Table 3, Figure S7).

In the first study of the quinonoid pigments of *D. setosum*, only echinochrome A was detected [34]; afterwards, Anderson et al. found that the pigments present in the Hawaiian sea urchin *D. setosum* are echinochrome A, spinochrome A and an unknown pigment [3]. Koltsova et al. isolated this unknown pigment, and identified it as echinochrome A methyl ether 7-ethyl-2,6-dihydroxy-3-methoxy-1,4-naphthoquinone (**22**) [35]. In addition, Koltsova et al. discovered echinochrome A and its methyl ether **22** in *D. savignyi*. Recently, Brasseur et al. discovered, in *D. savignyi* from Madagascar, echinochrome A as the main pigment and small amounts of spinochrome D-Iso 1 [12].

##### *Echinothrix* Genus

The total extracts of sea urchins of closely related species *Echinothrix calamaris* and *E. diadema* contained three main components, echinochrome A (**16**) and spinochromes E (**1**) and D (**3**) (Table 3, Figures S8 and S9). Two more compounds were present in the extract of *E. calamaris*, namely binaphthoquinone **11** and a pigment with *m/z* [M − H]^–^ of 247. Moore et al. previously isolated three pigments having the same molecular weight from the same species of sea urchin [36]. The absorption spectrum of the pigment found in this work turned out to be closest to that of compound **13** (Table 2). The extract of *E. diadema* also contained binaphthoquinone **11** and two minor pigments, dehydroechinochrome (**5**) and pigment **12**.

#### 2.3.4. Order Stomopneustoida

The total extract of the sea urchin *Stomopneustes variolaris*, the only representative of its genus, contained three compounds—echinochrome A (**16**) as the main pigment (81%), as well as insignificant amounts of its oxidation product **5** (approximately 9%) and spinochrome E (**1**) (approximately 10%) (Table 3, Figure S10). Previously, only echinochrome A was isolated from *S. variolaris* [37].

#### 2.3.5. Order Clypeasteroida

From the order Clypeasteroida, the composition of quinonoid pigments of flat sea urchins of four species was studied: *Echinarachnius parma* from the family Echinarachniidae, *Laganum decagonale* from the family Laganidae, and *Scaphechinus griseus* and *S. mirabilis* from the family Scutellidae.

The amphiboreal species *Echinarachnius parma* is distributed in the Pacific Ocean, from the Bering Sea to the Japanese Islands, and in the Atlantic Ocean it is common along the eastern coast of North America [38]. The total extract of *E. parma* from the Sea of Japan contained three main pigments, binaphthoquinone **11** (up to 52%), echinochrome A (**16**) (up to 25%) and spinochrome D (**3**) (up to 14%); as well as mirabiquinone (**9**) (up to 8%) and insignificant amounts of spinochrome E (**1**) (up to 5%), pigment **18** (up to 4%), and binaphthoquinone **14** (up to 2%) (Table 3, Figure S11). The composition and ratio of pigments of the extract of *E. parma* from the Sea of Japan were similar to those in *E. parma* from the Sea of Okhotsk studied previously; however, neither echinamines A and B were found, and pigment **18** was present [23].

Quinonoid pigments of *Laganum decagonale* were investigated here for the first time. Only the ethyl acetate extract of *L. decagonale* contained the following compounds: echinochrome A (**16**) (up to 45%), spinochromes C (**10**) (up to 37%) and E (**1**) (up to 17%) (Table 3, Figure S12).

Flat sea urchins *Scaphechinus mirabilis* are common in the Sea of Japan and the Sea of Okhotsk, on the Commander Islands on the eastern coast of Kamchatka, and live on sandy soils to a depth of 150 m, slightly sprinkled with sand, or burrow into the ground [39]. Earlier, only echinochrome A was found in *S. mirabilis* [40]. According to HPLC-MS analysis, the chloroform extract of *S. mirabilis* collected in the Sea of Japan contained predominantly echinochrome A (**16**), as well as small amounts of echinamines A (**19**) and B (**21**), binaphthoquinone **11** and spinochrome D (**3**) (Table 3, Figure S13). The ethyl acetate extract of *S. mirabilis* contained spinochrome D (**3**) and binaphthoquinones **9**, **11**, and **14** (Table 3, Figure S14).

Representative specimens of *S. griseus*, related to *S. mirabilis* species, live in the Sea of Japan and in the southern part of the Sea of Okhotsk, at depths from 1 to 50 m; similar to *S. mirabilis*, they prefer sandy soils. The quinonoid pigments of this species of flat sea urchins have not been previously studied. In the chloroform extract of *S. griseus* collected from the Sea of Japan, the main pigment was echinochrome A (**16**), similar to *S. mirabilis*, and spinochrome E (**1**) and binaphthoquinones **9** and **11** were also identified (Figure S15). The ethyl acetate extract of *S. griseus* contained spinochromes E (**1**) and D (**3**) and binaphthoquinones **11** and **14** (Figure S16).

#### 2.3.6. Order Spatangoida

The compositions of the quinonoid pigments of two heart-shaped sea urchins of various families of the Spatangoida order have been studied, collected from different climatic zones: *Echinocardium cordatum*, which lives in temperate latitudes, and the tropical species *Maretia planulata*.

*E. cordatum* is representative of heart-shaped sea urchins, at depths from littoral to 230 m in temperate latitudes of the Atlantic and Pacific oceans [41]. In the total extract of *E. cordatum* collected from the Sea of Japan, echinochrome A (**16**) (up to 88%), as well as spinochromes E (**1**) (approximately 8%) and D (**3**) (up to 4%) were mainly present (Table 3, Figure S17). Previously, only one pigment, echinochrome A, was found in this species of sea urchin [3].

The pigment composition of the tropical sea urchin *M. planulata* was studied here for the first time. The main pigment in the total extract of *M. planulata* was echinochrome A (**16**) (94%), and spinochrome E (**1**) (up to 6%) was also found (Table 3).

As a result, a number of conclusions can be drawn from Table 3.

It is well known that the composition of secondary metabolites in marine organisms may differ significantly, due to geographical, ecological, seasonal and gender differences, among other variables. Therefore, it is highly likely that reported differences in spinochrome compositions in sea urchins of the same species may be due to these variables.

Interestingly, in many cases, the spinochrome composition determined in this work differs from that published previously, which will be reasoned in the text (vide infra).

Already, fifty years has passed since Anderson et al. [3] summarized the data on the distribution of spinochromes in nearly 60 species of sea urchins, and found that echinochrome A and spinochromes A–E are the most common pigments found in sea urchins.

In 1966, the research group led by Richard E. Moore and Paul J. Scheuer isolated 11 new spinochromes, besides echinochrome A and spinochromes A–D, from the spines of two species of *Echinothrix* sea urchins, *E. diadema* and *E. calamaris*: 2-hydroxy-3-acetyl-naphthazarin, 2-hydroxy-6-ethyljuglone, 2-hydroxy-6-ethylnaphthazarin, naphthopurpurin, 2,7-dihydroxy-6-acetyljuglone, 2,7-dihydroxy-3-ethylnaphthazarin, 2,7-dihydroxynaphthazarin, 2,5-dihydroxy-3-ethylbenzoquinone, 2-hydroxy-6-acetylnaphthazarin, 2,3,7-trihydroxy-6-acetyljuglone, and 2,3,7-trihydroxy-6-ethyljuglone [36]. In 1968, the structure of the first pigment containing a four-carbon unit attached to a naphthoquinone system was described: 2-methyl-8-hydroxy-2H-pyrano[3,2-q]naphthazarin [42]. The structures of these compounds have been proven by spectral methods and synthesis; however, the nativeness of these compounds raises doubts, as for the past half century (and earlier), there has been no other report on the detection of these compounds in sea urchins. It is assumed that the juglone and naphthazarin derivatives described by Moore et al. may be artifacts of isolation, as, to isolate these pigments, the authors used extreme procedures, such as long-term separation on acidic silica gel and vacuum sublimation.

For over 15 years, the drug substance Echinochrome has been produced from the sea urchin *Scaphechinus mirabilis*; therefore, outcoming control of the content and composition of pigments in natural raw materials and analysis of accompanying impurities (their rate is not more than 2%) is performed in the finished product. Our experience shows that, at one of the stages of the technological process (vacuum sublimation stage), at least 10 degradation products of echinochrome A are formed, the structures of which were identified as derivatives of juglone (3,5,6,7-tetrahydroxy-2-ethyl-1,4-naphthoquinone; 2,5,6,7-tetrahydroxy-3-ethyl-1,4-naphthoquinone) and naphthazarin (2,5,8-trihydroxy-3-ethyl-1,4-naphthoquinone; 2,5,6,8-tetrahydroxy-3-ethyl-1,4-naphthoquinone (ethylmompain); 2,5,7,8-tetrahydroxy-3-ethyl-1,4-naphthoquinone (ethylisomompain); 2,3,5,8-tetrahydroxy-1,4-naphthoquinone (spinazarin); 6-ethyl-2,3,5,8-tetrahydroxy-1,4-naphthoquinone (ethylspinazarin) [19,43]. Similarly, 3-acetyl-2,7-dihydroxy-6-methylnaphthazarin was isolated from the test and spines of *M. nudus* after vacuum sublimation [25]. Moreover, the properties of the unprecedented 2,5-dihydroxy-3-ethylbenzoquinone, obtained from *Echinothrix* sea urchins as reported by Moore, fully correspond to the structure of benzoquinone forming as a product of the oxidative destruction of echinochrome A, isolated recently [44]. However, none of the aforementioned compounds were found in the original samples of sea urchin extracts for many years.

Therefore, Table S2 (Supplementary Material) is presented, containing quinonoid pigments with established structures found in various sea urchin species, as well as Table S3 (Supplementary Material), containing compounds that have been found at least once in sea urchins, but whose nativeness has not been proven.

Recently, Brasseur et al. in several reports have described three potentially new quinonoid pigments, spinochrome 252, echinamine 253 and spinochrome 282, along with novel spinochrome isomers: three for spinochrome D, three for spinochrome B, and two for spinochrome A [12,45,46,47]. Hypothetical molecular structures of isomers and new quinonoid pigments were suggested, based solely on their retention time and low-resolution mass spectra. At the same time, the authors provided in their words “accurate mass measurements”, *m/z* [M − H]^–^ of 237.00 for spinochrome D-Iso 1 and *m/z* [M − H]^–^ of 237.04 for spinochrome D-Iso 2 and spinochrome D-Iso 3. Hence, considering the difference between the mass values, these three compounds cannot have the same predicted formula (C_10_H_6_O_7_).

**Figure 2:**
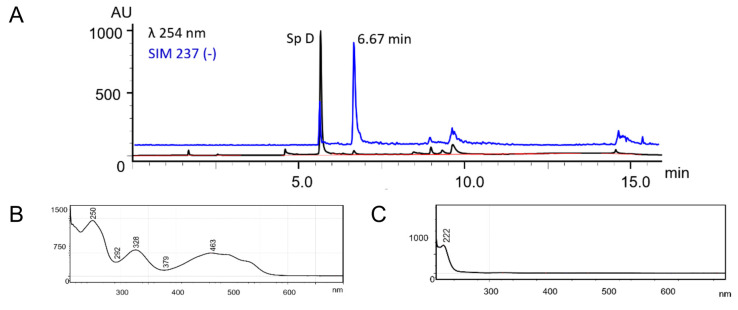
(**A**) HPLC chromatogram of one of the fractions obtained after chromatographic separation of the *S. mirabilis* ethyl acetate extract containing spinochrome D, together with the chromatogram obtained from the mass spectrometric (MS) detector in selected ion monitoring (SIM) mode with the targeted mass *m/z* [M − H]^–^ of 237. (**B**) Absorption spectrum of spinochrome D. (**C**) Absorption spectrum of the compound detected by SIM, with retention time 6.67 min.

Figure 2A shows the HPLC chromatogram of one fraction obtained after chromatographic separation of the *S. mirabilis* ethyl acetate extract containing spinochrome D [20], together with the chromatogram obtained from the MS-detector in selected ion monitoring (SIM) mode, with the targeted mass *m/z* [M − H]^–^ of 237. The compound with retention time 6.67 min has the same *m/z* [M − H]^–^ value (237) as spinochrome D; however, it does not have the quinonoid structure, as seen from Figure 2B,C which shows the absorption spectra of spinochrome D and the compound with retention time 6.67 min.

Another example is the compound 2,3,4,5,7-pentahydroxy-6-ethylinden-1-one, which also has *m/z* [M − H]^–^ at 237 and is a product of the oxidative destruction of echinochrome A (measured *m/z* [M − H]^–^ of 237.0397, compared to that calculated for C_11_H_9_O_6_, *m/z* [M − H]^–^ of 237.0405) [44]. Thus, considering the example of spinochrome D, we have doubts that reported by Brasseur et al. isomers of spinochromes A, B, and D have quinonoid nature.

Spinochromes easily fragment via the removal of H_2_O, CO, and CO_2_ [48,49]. In the experimental sections in previous reports by Brasseur et al. [12,45,46,47], it was indicated the mass spectrometric equipment used allows the obtaining of high-resolution mass spectra and of MS/MS fragmentation of quinonoid pigments. However, these data, as well as the absorption spectra, were not presented.

It is known that any assumption cited many times is eventually presented as fact. This is confirmed by the fact that the compounds claimed to be spinochrome A-Iso 2, spinochrome D-Iso 1 and spinochrome D-Iso 3, the structures of which were suggested in previous reports [12,45,46,47] from only their retention times and low-resolution mass spectra, are already included in other reviews in the list of known quinonoid metabolites of sea urchins [17,48].

Taking all of this into account, in addition to Tables S2 and S3 is presented Table S4 (Supplementary Material), containing quinonoid pigments found in sea urchins that have no established structure, but have at least absorption spectra and high-resolution mass spectra, and Table S5 (Supplementary Material), containing compounds found in sea urchins without proof of any quinonoid nature.

### 2.4. Total Quinonoid Pigments Content in Shells of Sea Urchins

The literature describes various methods for determining the quantitative content of pigments in sea urchins. Amarovich et al. calculated the total content of quinonoid pigments based on the ratio of the mass of the obtained extract to the mass of dry shells of sea urchins [50]. Powell et al. expressed the content of pigments in sea urchins as gallic acid equivalents, by the method of determination of total phenols [51]. A group of Belgian scientists used the MS method, using 2-hydroxy-1,4-naphthoquinone as an internal standard, to quantitatively evaluate the content of each pigment in the extract [12]. In this work, the total quantitative content of quinonoid pigments in sea urchins was determined spectrophotometrically, and expressed as the equivalent of echinochrome A (μg/g dry shell), as shown in Table 3.

Heart-shaped sea urchins *Echinocardium cordatum* and *Maretia planulata* were characterized by their low content of quinonoid pigments (38–42 µg/g), as reported for the boreal heart-shaped sea urchin *Brisaster latifrons* [23].

Among flat sea urchins, the content of quinonoid pigments was highly different between species. *L. decagonale* had a low content of quinonoid pigments (27.9 ± 8.8 μg/g). *S. griseus* were characterized by a moderate content of quinonoid pigments (87.3 ± 5.5 μg/g), and their related species *S. mirabilis* was characterized by a high content of pigments (1525.9 ± 93.4 μg/g). The content of pigments in samples of *E. parma* collected in the Sea of Japan (116.6 ± 8.6 μg/g) was almost four times higher compared to that of samples of *E. parma* from the Sea of Okhotsk (31.6 ± 0.6 μg/g) [23].

Regular sea urchins of the Strongylocentrotidae family *M. nudus* and *S. intermedius* had moderate pigment contents (89.9 ± 13.3 μg/g and 175.7 ± 11.3 μg/g, respectively), comparable to that of their related species *S. droebachiensis* [23]. In regular tropical sea urchins of the families Toxopneustidae, Cidaridae and Stomopneustidae, and in the genus *Echinothrix* from the Diadematidae family, the content of quinonoid pigments was moderate (70 to 140 μg/g). Sea urchins of the genus *Diadema* from the Diadematidae family differed, with a high content of quinonoid pigments (between 1129.9 ± 63.8 to 1267.1 ± 88.1 μg/g).

Thus, among the 16 species of Pacific sea urchins studied, the most promising sources of quinonoid pigments (in addition to *S. mirabilis*, which is already used to obtain the substance for Histochrome preparation) are sea urchins *D. savignyi* and *D. setosum*.

### 2.5. Quinonoid Pigments of CF of Sea Urchins

In the CF of sea urchins, various types of freely circulating cells have the common name of coelomocytes, and are divided into four subpopulations, the functions of which are still unclear: amoebocytes, red and colorless spherulocytes (morulocytes), and vibratil cells [52]. Coelomocytes are considered immune effectors of the sea urchin, due to their ability to respond to injuries, the invasion of viruses, germs, parasites and other factors. Coelomocytes respond to these interventions by phagocytosis, encapsulation and release of cytotoxic agents [53]. When studying the antimicrobial effect of the CF of the sea urchin *Echinus esculentus* towards marine bacteria, it was found that the main bactericidal agent of the CF is echinochrome A (**16**), which is contained in pigment granules of red spherulocytes [54]. In many later works devoted to the study of the functions of the CF of sea urchins, the authors refer to this study and indicate that red spherulocytes contain echinochrome A (**16**) [55,56,57]; however, chemical studies of the composition of the pigment granules have not been carried out. In several studies, the absorption spectra of CF extracts were presented as confirmation of the presence of echinochrome A (**16**) in spherulocytes [58].

We first conducted a study of the composition of the quinonoid pigments of the CF, from ten species of the following sea urchins: *Strongylocentrotus intermedius*, *S. pallidus*, *S. polyacanthus*, *Mesocentrotus nudus*, *Echinocardium cordatum*, *Scaphechinus mirabilis*, *Echinarachnius parma*, *Astropyga radiata*, *Diadema setosum* and *Echinothrix calamaris*.

**Figure 3:**
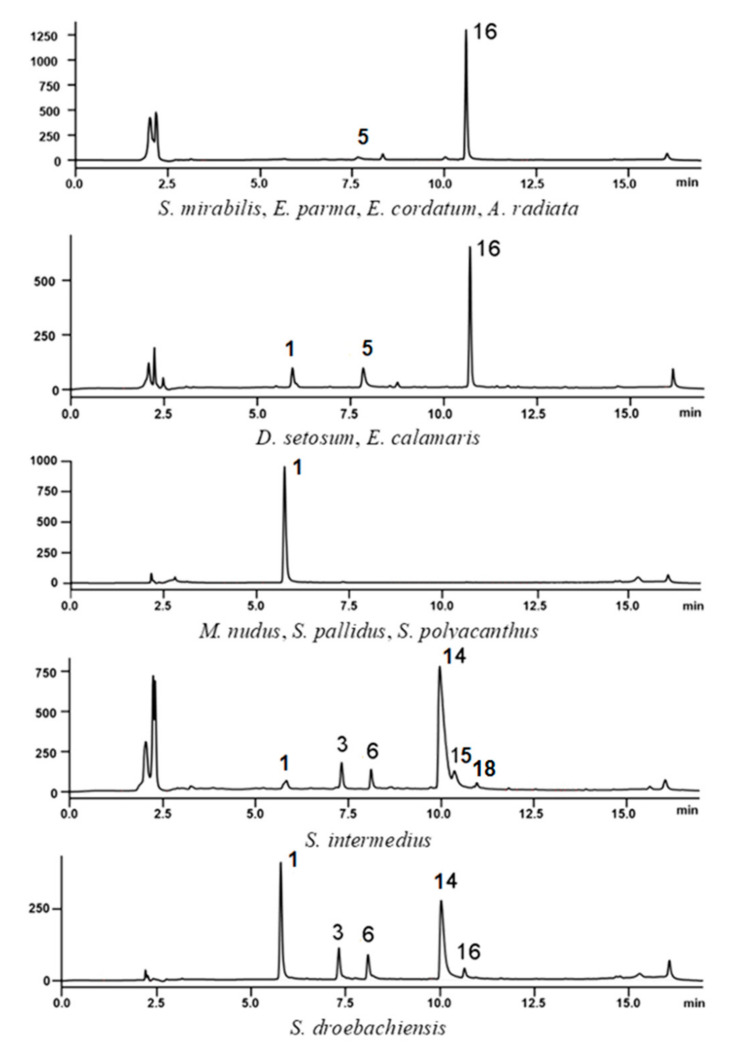
Typical HPLC profiles of extracts of the CF of Pacific sea urchins.

According to HPLC-MS analysis, the main pigment of the CF of sea urchins *S. mirabilis*, *E. parma*, *E. cordatum*, *A. radiata*, *D. setosum* and *E. calamaris* was echinochrome A (**16**), as well as minor amounts of its oxidation product dehydroechinochrome (**5**). In the CF of sea urchins *D. setosum* and *E. calamaris*, spinochrome E (**1**) was present (Figure 3) in addition to compounds **5** and **16**.

The absorption spectra of ethanol extracts of the CF of sea urchins *M. nudus*, *S. pallidus*, and *S. polyacanthus* differed from that of echinochrome A (Figure S18). When comparing the obtained data with the absorption spectra of standard samples of spinochromes, it was found that the spectrum of *M. nudus* CF extract coincided with the spectrum of spinochrome E (**1**), also confirmed by HPLC-MS analysis (Figure 3).

The absorption spectrum of *S. intermedius* CF extract is identical to the spectrum of echinochrome A (Figure S18); however, according to HPLC-MS analysis, the extract contained mainly binaphthoquinone **14**, as well as spinochromes E (**1**) and D (**3**), and three unidentified pigments **6**, **15** and **18** with *m/z* [M − H]^–^ of 535, 765 and 527, respectively (Figure 3). Based on this, it is clear that it is insufficient to use data on the absorption spectrum of the extract to determine the composition of pigments.

The composition of the quinonoid pigments of *S. droebachiensis* CF was close to that of *S. intermedius*; however, *S. droebachiensis* CF contained more spinochrome E (**1**), echinochrome A (**16**) was present, and pigments **15** and **18** were not detected (Figure 3). Previously, Hira et al. identified spinochromes C, D and E and binaphthoquinones **6** and **14** [48], in CF of *S. droebachiensis* from the coast of Tromsø, Norway.

Thus, it has been found that: the composition of the quinonoid pigments of the CF from sea urchins of different species may differ; they are not limited, as often believed, to echinochrome A; and they also correlate with the set of pigments present in the shells of the corresponding species of sea urchins.

It is shown in Table 4 that the content of quinonoid pigments in the CF of individual sea urchins varies within a fairly wide range. The distribution of the values of spinochrome content in the CF of individual animals was highly heterogeneous, and did not form a maximum peak as in the previous report [54]. In this study, sea urchins were not distinguished by health status, age, sex and other characteristics, as this is a difficult task.

### 2.6. Quinonoid Pigments of Eggs and Embryos of Sea Urchins

Previously, Koltsova et al., using thin layer chromatography (TLC), found only one pigment, echinochrome A, in extracts of non-fertilized eggs, embryos at the gastrula stage, and pluteus of sand dollar *Scaphechinus mirabilis* [59]. Recent mass spectrometric studies have shown that the pigment composition in the eggs and embryos of this sea urchin is more diverse. Thus, Drozdov et al., using the enzymatic method of pigment extraction, which does not require sample preparation of the crude extract, and analyzing the mass spectra of anions obtained by matrix-assisted laser desorption/ionization (MALDI-TOF-MS), found that pigment granules of *S. mirabilis* eggs contain spinochrome E, and a small amount of spinochrome D; while echinochrome A and spinochrome D have also been found in shells of adult sea urchins [60].

In acidic ethanol extracts of *S. mirabilis* eggs, spinochrome E and a small amount of spinochrome D were also found using ESI-MS; however, in shells of this sea urchin, in addition to echinochrome A and spinochrome D, echinamines A and B and binaphthoquinones were identified (Table 3). For the first time, red pigment granules appear in *S. mirabilis* embryos at the stage of early gastrula. At the stage of pluteus, the number of pigment granules increases significantly [61].

According to our data, on days 3 and 21 of the development of the *S. mirabilis* pluteus, the pigment composition remains the same: echinochrome A and spinochrome E, and trace amounts of spinochrome D (Figure S19). The same composition of pigments was found by Ageenko et al. in 3-day blastula-derived primary cell cultures of *S. mirabilis* [62].

In another species of sea urchins of the order Clypeasteroida *E. parma* in a jelly-like egg membrane, a wide range of quinonoid pigments with compositions similar to those of the shell pigment was found, mainly binaphthoquinones **14** and **6** and spinochrome D (**3**), and small contents of echinochrome A (**16**) and spinochrome E (**1**) were identified (Figure S20).

From this, it follows that, in the same species of sea urchins, the composition of quinonoid pigments in the shell epidermis, red spherulocytes of the CF, the jelly-like egg membrane, and in developing embryos may be different, as they perform different functions in the development and the preservation of the species, but it is usually more diverse in the epidermis of the shell of adult sea urchins, as this region is most susceptible to aggressive environmental influences.

## 3. Materials and Methods 

### 3.1. Materials

HPLC-grade water and acetic acid were purchased from Panreac Quimica (Barcelona, Spain). MeCN (grade 0) was sourced from Cryochrom (Saint Petersburg, Russia). Other solvents used in this study were of analytical grade. Ethanol (LLC “Bifarm”, Moscow, Russia) was distilled prior to use. The standard sample of echinochrome A (registration number P N002362/02-2003) was produced by G.B. Elyakov Pacific Institute of Bioorganic Chemistry (Vladivostok, Russia). Mompain was isolated as an impurity of the drug substance echinochrome A [19]. Mirabiquinone [20] and echinamines A and B [21] were isolated previously from *Scaphechinus mirabilis*. Dehydroechinochrome [22] and echinochrome A monomethyl ethers **20** and **22** [23] were obtained synthetically from echinochrome A. Spinochromes A, B, C and E and spinamine E were isolated from *St. pallidus* and *M. nudus* [10]. Spinochrome D, 7,7′-anhydroethylidene-6,6′-bis(2,3,7-trihydroxynaphthazarin), and ethylidene-3,3′-bis(2,6,7-trihydroxynaphthazarin) were isolated from *Astropyga radiata* [23].

### 3.2. HPLC-DAD-MS Analysis

HPLC-DAD-MS was performed using a system consisting of a CBM-20A system controller (Shimadzu USA Manufacturing Inc., Canby, OR, USA), two LC-20 CE pumps (Shimadzu USA Manufacturing Inc., Canby, OR, USA), a DGU-20A3 degasser (Shimadzu Corp., Kyoto, Japan), a SIL-20A autosampler (Shimadzu USA Manufacturing Inc., Canby, OR, USA), a diode-matrix SPD-M20A (Shimadzu USA Manufacturing Inc., Canby, OR, USA), and mass-spectrometric detector LCMS-2020 (Shimadzu Corp., Kyoto, Japan). The separation was carried out on a Discovery HS C18 column (150 × 2.1 mm, 3 µm particle size, Supelco, Bellefonte, PA, USA) with a Supelguard Ascentis C18 pre-column (2 × 2.1 mm, 3 µm particle size, Supelco, Bellefonte, PA, USA) using a binary gradient of H_2_O (A): MeCN (B) with the addition of 0.2% AcOH, at a flow rate of 0.2 mL/min and column temperature of 40 °C. The gradient was as follows: 0–6 min, 10–40% (B); 6–11 min, 40–100% (B); 11–12 min, 100% (B), 12–13 min, 100–10% (B); and 13–17 min, 10% (B). The chromatograms were recorded at 254 nm. Mass spectra were taken in ESI mode at atmospheric pressure, recording negative ions (1.50 kV) in the *m/z* range of 100–900, with N_2_ as drying gas (10 L/min) and N_2_ nebulizer gas flow (1.5 L/min), temperature for the curved desolvation line (CDL) at 200 °C and for the heat block at 250 °C, and interface voltage of 3.5 kV. Prior to analysis, samples were filtered through a 0.2 µm PTFE syringe filter. The injection volume was 3 µL.

### 3.3. HPLC Method Validation

The linearity of the method was established by using methanolic solutions of standard samples of echinochrome A, spinochromes D and E, and 7,7′-anhydroethylidene-6,6′-bis (2,3,7-trihydroxy-naphthazarin) at concentrations of 50–1500 ng/mL. Each sample was injected at least in triplicate. Calibration curves were constructed as a linear regression analysis of the peak area versus concentration. The limits of detection (LOD) and quantification (LOQ) of standard samples were calculated as concentrations at which the signal-to-noise ratio is below 3 and 10, respectively. The accuracy of the method was established by recovery studies of standard samples (100–1300 ng/mL), and this data is provided in Table S1. Accuracy was expressed as relative standard deviation (RSD) and recovery (%). Selectivity was confirmed via peak purity studies using a DAD detector.

### 3.4. Animal Material

Sea urchins of the Sea of Japan were harvested by scuba divers at depths of 1–20 m in Troitsa Bay, at the Marine Experimental Station (Risovaya Pad, Primorsky Krai, 692707) (Table 5). Taxonomic identification was provided by Prof. A.L. Drozdov of the A.V. Zhirmunsky Institute of Marine Biology, of the Far Eastern Branch of the Russian Academy of Sciences. Voucher specimens of the above-mentioned sea urchins were deposited in the collection of the Laboratory of Chemistry of Natural Quinonoid Compounds, of the G.B. Elyakov Pacific Institute of Bioorganic Chemistry, Vladivostok, Russia.

Samples of sea urchins of the South China Sea were collected by both dredging and scuba divers, during the 45th and 49th scientific cruises of R/V Academic Oparin, in June 2013 and November 2016, respectively. The details are presented in Table 5. The species of sea urchins were identified by Dr. Nguyen Thi My Ngan, Department of Museum, Institute of Oceanography, Nhatrang, Vietnam, and deposited in the Nhatrang Institute of Technology Research and Application, Nhatrang, Vietnam.

Immediately after collection of the animal material, the internal organs of the sea urchins were removed; shells and spines were crushed (except for *S. mirabilis, S. griseus, E. parma*, and *L. decagonale*, which were frozen whole) and stored in a −20 °C freezer prior to extraction.

The CF of sea urchins *M. nudus*, *S. intermedius*, *S. pallidus*, *S. polyaccanthus*, *S. droebachiensis* and *E. cordatum* was withdrawn through the peristomial membrane using a syringe, into a 50 mL plastic tube, according to [58]. CF from *S. mirabilis* and *E. parma* was collected as described in [63]. No anti-coagulant was used; CF from each sea urchin was left in plastic tubes for clotting for 2 h at room temperature. Clots were separated from serum by centrifugation (3000 rpm, 10 °C, 20 min), and stored in a −20 °C freezer prior to extraction.

### 3.5. Quinonoid Pigments Extraction

The defrosted shells and spines (50 g) of each sea urchin sample were extracted in three replicates, using 70% ethanol containing 10% H_2_SO_4_ at room temperature for 4 h. The acidified EtOH extract (200 mL) was centrifuged (3000 g, 20 min, 10 °C), and the supernatant was concentrated in vacuo at 55 °C. The viscous residue was diluted with an equal volume of distilled water, and subsequently extracted with chloroform and ethyl acetate. After evaporation of the solvents, the chloroform and ethyl acetate extracts were stored in a freezer at −20 °C prior to HPLC-DAD/MS analysis.

Defrosted CF clots were extracted with 3 mL EtOH containing 5% H_2_SO_4_, at room temperature for 5 h. The EtOH extract was centrifuged (3000 g, 10 min) using a High Speed Centrifuge type 310b (Mechanika Precyzyjna, Warsaw, Poland), and supernatant aliquots of 1 mL were filtered through 0.2 µm syringe filters, and used for HPLC-DAD-MS analysis and total PHNQ content determination.

### 3.6. Total Quinonoid Pigments Content

The content of quinonoid pigments in sea urchin shells was determined spectrophotometrically using a UV-mini 1240 (Shimadzu Corp., Kyoto, Japan), and expressed as µg of echinochrome A equivalent per g of dry shells, as described elsewhere [23] (details in Supplementary Material).

Similarly, the optical density of a solution of 200–500 μL of CF extract in 2.5 mL of ethanol was measured; the content of quinonoid pigments was calculated from the calibration curve and expressed as the equivalent of echinochrome A to the volume of CF of sea urchin (μg/mL).

### 3.7. Statistical Analysis

All measurements for quinonoid pigments content determination both using HPLC-DAD-MS and spectrophotometer were conducted at least in five replicates, which suggests results from at least five extractions of sea urchin samples. Data were presented as means ± standard errors. Statistical analysis was performed using STATISTICA v.10, Stat. Soft Inc. software.

## 4. Conclusions

The developed HPLC-DAD-MS method and the obtained properties of spinochromes will be highly useful, not only for determination of composition and content of quinonoid pigments in various sea urchin samples, but also for monitoring the quality of drugs and food additives based on sea urchin pigments, in addition to studying their stability.

Using the validated HPLC-DAD-MS method, composition and content of quinonoid pigments of 16 species of sea urchins collected in various regions of the Pacific Ocean over several years was investigated. Based on these results, an attempt has been made to analyze the structural diversity of spinochromes described in the literature, in order to distinguish compounds with accurately established structures from unknown and likely non-existent compounds. Firstly, a study was conducted of the composition of quinonoid pigments in the CF of ten species of sea urchins, and it was found that this composition correlates with the set of pigments present in the shells of the corresponding sea urchin species. The composition of quinonoid pigments of *E. parma* jelly-like egg membrane was reported first, and appeared to be similar to the shell pigment composition. In the case of the sea urchin *Scaphechinus mirabilis*, it was shown that the compositions of pigment granules in the shell epidermis, CF, egg membrane, developing embryos and pluteus are different. It is assumed that different spinochromes each perform different functions in the development and the life support of sea urchins, explaining the changes in pigment composition over the entire life cycle.

It is expected that the accumulated reliable information on the composition and quantity of spinochromes, at various stages of the sea urchin life cycle, will assist biologists studying developmental problems, as well as specialists studying the immune system and the influence of environmental factors on it. Ultimately, as quinonoid pigments of sea urchins exhibit pronounced biological effects, this work may help to identify new molecular targets and give preconditions for the creation of new drugs.

## Figures and Tables

**Table 1:** Validation parameters of the main components of sea urchin extracts at λ 254 nm.

Standard Sample	Equation of Peak Area Versus Concentration	R^2^	LOD * (ng/mL)	LOQ ** (ng/mL)
**1**	y = 7.3003x − 0.1007	0.9992	48	159
**3**	y = 6.5882x − 0.2588	0.9990	40	134
**11**	y = 4.5986x − 0.4385	0.9984	72	240
**16**	y = 9.5797x − 0.2320	0.9991	22	72

* LOD: limit of detection; ** LOQ: limit of quantification.

**Table 2:** HPLC-MS parameters of quinonoid pigments of sea urchins.

No	Rt, min	*m/z* [M − H]^–^	λ_max_, nm	Formula	Compound
1	5.56	253	264, 350, 478	C_10_H_6_O_8_	Spinochrome E
2	6.67	252	275, 370, 473	C_10_H_7_NO_7_	Spinamine E
3	7.09	237	251, 327, 463	C_10_H_6_O_7_	Spinochrome D
4	7.45	221	265, 320, 390, 471	C_10_H_6_O_6_	Spinochrome B
5	7.53	299	256, 321, 391	C_12_H_12_O_9_	Dehydroechinochrome
6	8.11	535	260, 333, 394, 473	-	Not identified
7	8.40	221	270, 315, 515, 559	C_10_H_6_O_6_	Mompain
8	8.49	262	272, 319, 511	C_12_H_9_NO_6_	Acetylaminotrihydroxynaphthoquinone
9	8.86	483	264, 325, 452	C_22_H_12_O_13_	Mirabiquinone
10	9.77	279	290, 456	C_12_H_8_O_8_	Spinochrome C
11	9.83	483	265, 316, 470	C_22_H_12_O_13_	7,7′-Anhydroethylidene-6,6′-bis(2,3,7-trihydroxynaphthazarin)
12	10.14	499	264, 304, 408, 469, 532	-	Not identified
13	10.40	247	219, 268, 299	C_12_H_8_O_6_	2-Acetyl-3-hydroxynaphthazarin
14	10.42	501	254, 339, 471	C_22_H_14_O_14_	Ethylidene-3,3′-bis(2,6,7-trihydroxynaphthazarin)
15	10.46	765	217, 273, 344, 473	-	Not identified
16	10.62	265	254, 338, 471	C_12_H_10_O_7_	Echinochrome A
17	10.64	263	266, 312, 508	C_12_H_8_O_7_	Spinochrome A
18	11.06	527	260, 335, 495, 542	-	Not identified
19	11.35	264	278, 352, 477	C_12_H_11_NO_6_	Echinamine A
20	11.56	279	252, 330, 491, 525	C_13_H_12_O_7_	7-Ethyl-3,5,6,8-tetrahydroxy-2-methoxy-1,4-naphthoquinone
21	11.66	264	274, 352, 477	C_12_H_11_NO_6_	Echinamine B
22	11.73	279	256, 332, 474, 497, 538	C_13_H_12_O_7_	7-Ethyl-2,5,6,8-tetrahydroxy-3-methoxy-1,4-naphthoquinone

**Table 3:** Occurrence and content of quinonoid pigments in the shells of sea urchins.

Family Species	Content of Main Spinochromes, % of Pigment Sum						Other Pigments, %	Total PHNQ Content, µg/g
	Ech A (16)	A (17)	B (4)	C (10)	D (3)	E (1)		
**REGULAR SEA URCHINS**								
**Order Camarodonta**								
**Strongylocentrotidae** *Mesocentrotus nudus*	21.0 ± 3.7	10.5 ± 2.2	2.1 ± 0.3	3.5 ± 2.2	2.1 ± 0.6	54.2 ± 13.1	2 (2.2 ± 1.8), 18 (4.4 ± 3.5)	89.9 ± 13.3
*Strongylocentrotus intermedius*		3.3 ± 0.3	4.0 ± 1.3	9.2 ± 1.5	16.5 ± 5.7	3.1 ± 1.6	9 (3.9 ± 0.6), 11 (51.3 ± 7.7), 18 (8.8 ± 5.2)	175.7 ± 11.3
**Toxopneustidae** *Toxopneustes pileolus*	79.5 ± 5.3		3.7 ± 0.2	4.8 ± 1.7		6.4 ± 2.1	14 (5.6 ± 2.2)	90.2 ± 7.3
*Tripneustes gratilla*		26.1 ± 4.3				67.2 ± 11.8	2 (4.7 ± 2.1), 7 (2.0 ± 1.8)	93.3 ± 6.8
**Order Cidaroida**								
**Cidaridae** *Phyllacanthus imperialis*		74.7 ± 5.7		23.1 ± 3.8			8 (3.2 ± 0.2)	87.3 ± 5.5
**Order Diadematoida**								
**Diadematidae** *Diadema savignyi*	80.2 ± 6.9				1.1 ± 0.7	3.5 ± 2.2	5 (3.3 ± 0.3), 11 (2.9 ± 2.5), 12 (3.0 ± 0.9) 20 (3.4 ± 0.1), 22 (2.6 ± 0.2)	1129.9 ± 63.8
*Diadema setosum*	92.1 ± 4.2					3.8 ± 2.2	20 (4.1 ± 0.3)	1267.1 ± 88.1
*Echinothrix calamaris*	65.3 ± 9.6				6.2 ± 1.2	13.9 ± 3.7	11 (2.9 ± 1.9), 13 (3.5 ± 0.3), 14 (8.2 ± 0.3)	138.8 ± 7.3
*Echinothrix diadema*	39.9 ± 11.3				9.7 ± 2.9	7.4 ± 1.5	5 (1.7 ± 1.5), 11 (36.9 ± 8.8), 12 (1.4 ± 0.3), 15 (3.0 ± 1.4)	116.6 ± 5.9
**Order Stomopneustoida**								
**Stomopneustidae** *Stomopneustes variolaris*	81.4 ± 9.1					10.3 ± 6.8	5 (9.3 ± 0.8)	66.7 ± 5.1
**IRREGULAR SEA URCHINS**								
**Order Clypeasteroida**								
**Echinarachniidae** *Echinarachnius parma*	14.6 ± 5.2				13.3 ± 1.9	1.3 ± 0.7	9 (3.0 ± 1.3), 11 (61.7 ± 18.0), 18 (6.1 ± 0.6)	116.6 ± 7.4
**Laganidae** *Laganum decagonale*	32.4 ± 7.3			28.3 ± 8.1	11.7 ± 4.7	27.6 ± 4.3		27.9 ± 8.8
**Scutellidae** *Scaphechinus mirabilis*	89.1 ± 8.7				1.8 ± 0.9		9 (2.0 ± 0.2), 11 (2.3 ± 1.1), 14 (1.8 ± 0.4), 19 (1.1 ± 0.1), 21 (1.9 ± 0.1),	1525.9 ± 93.4
*Scaphechinus griseus*	28.6 ± 12.1				18.3 ± 6.2	4.3 ± 0.4	11 (45.5 ± 13.4), 14 (3.3 ± 0.3)	87.3 ± 5.5
**Order Spatangoida**								
**Loveniidae** *Echinocardium cordatum*	78.1 ± 6.7				1.7 ± 0.2	20.2 ± 10.9		37.8 ± 6.8
**Maretiidae** *Maretia planulata*	94.3 ± 5.4					5.7 ± 0.9		42.6 ± 3.7

**Table 4:** Content of quinonoid pigments in the CF of sea urchins (*n* is the number of individuals).

Species	*E. cordatum* (*n* = 63)	*E. parma* (*n* = 77)	*M. nudus* (*n* = 61)	*S. intermedius* (*n* = 39)	*S. mirabilis* (*n* = 85)
Quinonoid pigments content,µg/mL	8–38	4–31	14–103	5–27	10–93

**Table 5:** List of the collected sea urchin samples.

Species	Collection Area and Period	Coordinates	Depth, m
**South China Sea**			
*Astropyga radiata* (Leske, 1778)	Nha Trang Bay, June 2014	12°11′49″ N, 109°15′55″ E	12
*Diadema savignyi* (Audouin, 1829)	Hon Tre I., July 2013	12°13′58.5″ N, 109°14′02.0″ E	5
*Diadema setosum* (Leske, 1778)	Hon Tre I., July 2013 Ly Son I., November 2016	12°13′58.5″ N, 109°14′02.0″ E 15°22′06.9″ N 109°06′62.5″ E	5 15
*Echinothrix calamaris* (Pallas, 1774)	Nha Trang Bay, July 2013 Ly Son I., November 2016	09°55′26.7″ N, 104°01′57.7″ E 15°22′06.9″ N 109°06′62.5″ E	8 15
*Echinothrix diadema* (Linnaeus, 1758)	Nha Trang Bay, July 2013 Con Co I., November 2016	12°11′47.3″ N, 109°16′01.7″ E 17°08′95.3″ N 107°20′56.5″ E	10 10
*Laganum decagonale* (Blainville, 1827)	Con Co I., November 2016	17°02′5″ N 107°36′2″ E	55
*Maretia planulata* (Lamarck, 1816)	Hon Tre I., July 2013	12°11′47.3″ N, 109°16′01.7″ E	17
*Phyllacanthus imperialis* (Lamarck, 1816)	Hon Tre I., July 2013	12°11′47.3″ N, 109°16′01.7″ E	11
*Stomopneustes variolaris* (Lamarck, 1816)	Hon Tre I., July 2013	12°13′58.5″ N, 109°14′02.0″ E	8
*Toxopneustes pileolus* (Lamarck, 1816)	Nam Du I., July 2013 Ly Son I., November 2016	09°44′43.8″ N, 104°21′72.6″ E 15°22′06.9″ N 109°06′62.5″ E	7 9
*Tripneustes gratilla* (Linnaeus, 1758)	Ca Na Bay, July 2013 Cu Lao Cham I., November 2016	11°13′92.4″ N, 108°50′28.0″ E 15°54′14.4″ N 108°32′03.0″ E	20 7
**Sea of Japan**			
*Echinarachnius parma* (Lamarck, 1816)	Troitsa Bay, August 2014–2020	42°37′29.8″ N, 131°07′29.1″ E	1–16
*Echinocardium cordatum* (Pennant, 1777)	Troitsa Bay, August 2014–2020	42°37′29.8″ N, 131°07′29.1″ E	1–20
*Mesocentrotus nudus* (A. Agassiz, 1864)	Troitsa Bay, August 2014–2020	42°37′29.8″ N, 131°07′29.1″ E	1–12
*Scaphechinus mirabilis* (A. Agassiz, 1864)	Troitsa Bay, August 2014–2020	42°37′29.8″ N, 131°07′29.1″ E	1–12
*Scaphechinus griseus* (Mortensen, 1927)	Troitsa Bay, August 2014–2020	42°37′29.8″ N, 131°07′29.1″ E	1–17
*Strongylocentrotus intermedius* (A. Agassiz, 1864)	Troitsa Bay, August 2014–2020	42°37′29.8″ N, 131°07′29.1″ E	1–10

## Data Availability

The data presented in this study are fully available in the main text and supplementary materials of this article.

## Supplementary Materials

The following are available online at https://www.mdpi.com/1660-3397/19/1/21/s1. Table S1. Accuracy and reproducibility of the quantification of standard samples spinochrome E (1), spinochrome D (3), 7,7′-anhydroethylidene-6,6′-bis(2,3,7-trihydroxynaphthazarin) (11), and echinochrome A (16) using HPLC method; Table S2. Quinonoid pigments with established structure, found in various sea urchin species; Table S3. Quinonoid pigments with established structure that have been found at least once in sea urchins, but their nativeness has not been proven; Table S4. Quinonoid pigments found in sea urchins, that have no established structure; Table S5. Compounds found in sea urchins without proof of their quinonoid nature; Figure S1. Typical HPLC profile of Strongylocentrotus intermedius total extract; Figure S2. Typical HPLC profile of Toxopneustes pileous total extract; Figure S3. Typical HPLC profile of Tripneustes gratilla CHCl3 extract; Figure S4. Typical HPLC profile of Tripneustes gratilla EtOAc extract; Figure S5. Typical HPLC profile of Phyllacanthus imperialis CHCl3 extract; Figure S6. Typical HPLC profile of Diadema savignyi total extract; Figure S7. Typical HPLC profile of Diadema setosum total extract; Figure S8. Typical HPLC profile of Echinothrix calamaris total extract; Figure S9. Typical HPLC profile of Echinothrix diadema total extract; Figure S10. Typical HPLC profile of Stomopneustes variolaris total extract; Figure S11. Typical HPLC profile of Echinarachnius parma total extract; Figure S12. Typical HPLC profile of L. decagonale total extract; Figure S13. Typical HPLC profile of Scaphechinus mirabilis CHCl3 extract; Figure S14. Typical HPLC profile of Scaphechinus mirabilis EtOAc extract; Figure S15. Typical HPLC profile of Scaphechinus griseus CHCl3 extract; Figure S16. Typical HPLC profile of Scaphechinus griseus EtOAc extract; Figure S17. Typical HPLC profile of Echinocardium cordatum total extract; Figure S18. Absorption spectra (ethanol): (A) echinochrome A (16) and spinochrome E (1) in comparison with the spectrum of the M. nudus coelomic fluid (CF) extract; (B) echinochrome A (16) in comparison with the spectrum of S. intermedius CF; Figure S19. HPLC profile of Scaphechinus mirabilis 3-day and 21-day pluteus; Figure S20. HPLC profile of Echinarachnius parma jelly-like egg membrane pigment granules.
