# Supplementary material for: Spinochrome Identification and Quantification in Pacific Sea Urchin Shells, Coelomic Fluid and Eggs Using HPLC-DAD-MS
Source: Mar Drugs. 2021 Jan 6;19(1):21. doi: 10.3390/md19010021 (PMC7825409; doi:10.3390/md19010021)
Supplement: Supplementary file 1 [file marinedrugs-19-00021-s001.pdf]

# - Supporting Information –

Spinochromes identification and quantification in Pacific sea urchins shells, coelomic fluid, and eggs using HPLC-DAD-MS

Elena A. Vasileva [1,*], Natalia P. Mishchenko [1], Van T. T. Tran [2], Hieu M. N. Vo [2], and Sergey A. Fedoreyev [1]

[1] Laboratory of the Chemistry of Natural Quinonoid Compounds, G.B. Elyakov Pacific Institute of Bioorganic Chemistry, Far Eastern Branch of Russian Academy of Sciences, 690022, Vladivostok, Russia; vasilieva_el_an@mail.ru (E.A.V.); mischenkonp@mail.ru (N.P.M.); fedoreev-s@mail.ru (S.A.F.)

[2] Nhatrang Institute of Technology Research and Application, VAST, Khanh Hoa, Vietnam; vanvvlnt@yahoo.com.vn (V.T.T.T.); nhuhieu@nitra.vast.vn (H.M.N.V.)

* Correspondence: vasilieva_el_an@mail.ru; Tel.: +7-9025272055

TABLE OF CONTENTS:

Table S1. Accuracy and reproducibility of the quantification of standard samples spinochrome E (**1**), spinochrome D (**3**), 7,7'-anhydroethylidene-6,6'-bis(2,3,7-trihydroxynaphthazarin) (**11**), and echinochrome A (**16**) using HPLC method. 3

Figure S1. Typical HPLC profile of *Strongylocentrotus intermedius* total extract 4

Figure S2. Typical HPLC profile of *Toxopneustes pileous* total extract 4

Figure S3. Typical HPLC profile of *Tripneustes gratilla* $CHCl_3$ extract 5

Figure S4. Typical HPLC profile of *Tripneustes gratilla* EtOAc extract 5

Figure S5. Typical HPLC profile of *Phyllacanthus imperialis* $CHCl_3$ extract 6

Figure S6. Typical HPLC profile of *Diadema savignyi* total extract 6

Figure S7. Typical HPLC profile of *Diadema setosum* total extract 7

Figure S8. Typical HPLC profile of *Echinothrix calamaris* total extract 7

Figure S9. Typical HPLC profile of *Echinothrix diadema* total extract 8

Figure S10. Typical HPLC profile of *Stomopneustes variolaris* total extract 8

Figure S11. Typical HPLC profile of *Echinarachnius parma* total extract 9

Figure S12. Typical HPLC profile of *L. decagonale* total extract 9

Figure S13. Typical HPLC profile of *Scaphechinus mirabilis* $CHCl_3$ extract 10

Figure S14. Typical HPLC profile of *Scaphechinus mirabilis* EtOAc extract 10

Figure S15. Typical HPLC profile of *Scaphechinus griseus* $CHCl_3$ extract 11

Figure S16. Typical HPLC profile of *Scaphechinus griseus* EtOAc extract 11

Figure S17. Typical HPLC profile of *Echinocardium cordatum* total extract 12

Table S2. Quinonoid pigments with established structure, found in various sea urchin species 13

Table S3. Quinonoid pigments with established structure that have been found at least once in sea urchins, but their nativeness has not been proven. 14

Table S4. Quinonoid pigments found in sea urchins, that have no established structure. 15

Table S5. Compounds found in sea urchins without proof of their quinonoid nature. 16

Figure S18. Absorption spectra (ethanol): **A**) echinochrome A (**16**) and spinochrome E (**1**) in comparison with the spectrum of the *M. nudus* coelomic fluid (CF) extract; **B**) echinochrome A (**16**) in comparison with the spectrum of *S. intermedius* CF. 17

Figure S19. HPLC profile of *Scaphechinus mirabilis* 3-day and 21-day pluteus. 17

Figure S20. HPLC profile of *Echinarachnius parma* jelly-like egg membrane pigment granules 18

Total Quinonoid Pigments Content 18

References 19

Table S1. Accuracy and reproducibility of the quantification of standard samples spinochrome E (**1**), spinochrome D (**3**), 7,7'-anhydroethylidene-6,6'-bis(2,3,7-trihydroxynaphthazarin) (**11**), and echinochrome A (**16**) using HPLC method.

**Spinochrome E (1)**	**Level 1 (100 ng/ml)**	**Accuracy, %**	**Level 2 (350 ng/ml)**	**Accuracy, %**	**Level 3 (700 ng/ml)**	**Accuracy, %**
Sample 1	108	108.0	329	94.0	728	104.0
Sample 2	93	93.0	346	98.8	716	102.3
Sample 3	100	100.0	359	102.6	687	98.1
Sample 4	98	98.0	351	100.3	703	100.4
$\bar{X}$*	99.8		346.3		708.5	
SD**	6.2		12.7		17.6	
S_r***, %	6.2		3.7		2.5	
**Spinochrome D (3)**	**Level 1 (200 ng/ml)**	**Accuracy, %**	**Level 2 (400 ng/ml)**	**Accuracy, %**	**Level 3 (700 ng/ml)**	**Accuracy, %**
Sample 1	193	96.5	422	105.5	669	95.6
Sample 2	201	100.5	403	100.7	723	103.3
Sample 3	211	105.5	388	97.0	711	101.6
Sample 4	198	99.0	392	98.0	681	97.3
$\bar{X}$*	200.7		401.2		696	
SD**	7.6		15.2		25.2	
S_r***, %	3.8		3.8		3.6	
**7,7'-anhydroethylidene-6,6'-bis(2,3,7-trihydroxynaphthazarin) (11)**	**Level 1 (250 ng/ml)**	**Accuracy, %**	**Level 2 (700 ng/ml)**	**Accuracy, %**	**Level 3 (1300 ng/ml)**	**Accuracy, %**
Sample 1	261	104.4	683	97.6	1403	107.9
Sample 2	238	95.2	712	101.7	1362	97.3
Sample 3	259	103.6	734	104.8	1278	98.3
Sample 4	247	98.8	691	98.7	1316	101.2
$\bar{X}$*	251.2		705		1339.7	
SD**	10.8		22.8		54.4	
S_r***, %	4.3		3.2		4.0	
**Echinochrome A (16)**	**Level 1 (100 ng/ml)**	**Accuracy, %**	**Level 2 (250 ng/ml)**	**Accuracy, %**	**Level 3 (500 ng/ml)**	**Accuracy, %**
Sample 1	105	105.0	238	95.2	512	102.4
Sample 2	100	100.0	248	99.2	518	103.6
Sample 3	98	98.0	253	101.2	503	100.6
Sample 4	103	103.0	262	104.8	509	101.8
$\bar{X}$*	101.5		250.3		510.5	
SD**	3.1		10.2		6.2	
S_r***, %	3.06		4.0		1.2	

*$\bar{X}$ – mean; **SD – standard deviation; ***S_r – relative standard deviation, %

All the unmarked peaks throughout the file do not have characteristic quinonoid absorption spectrum.

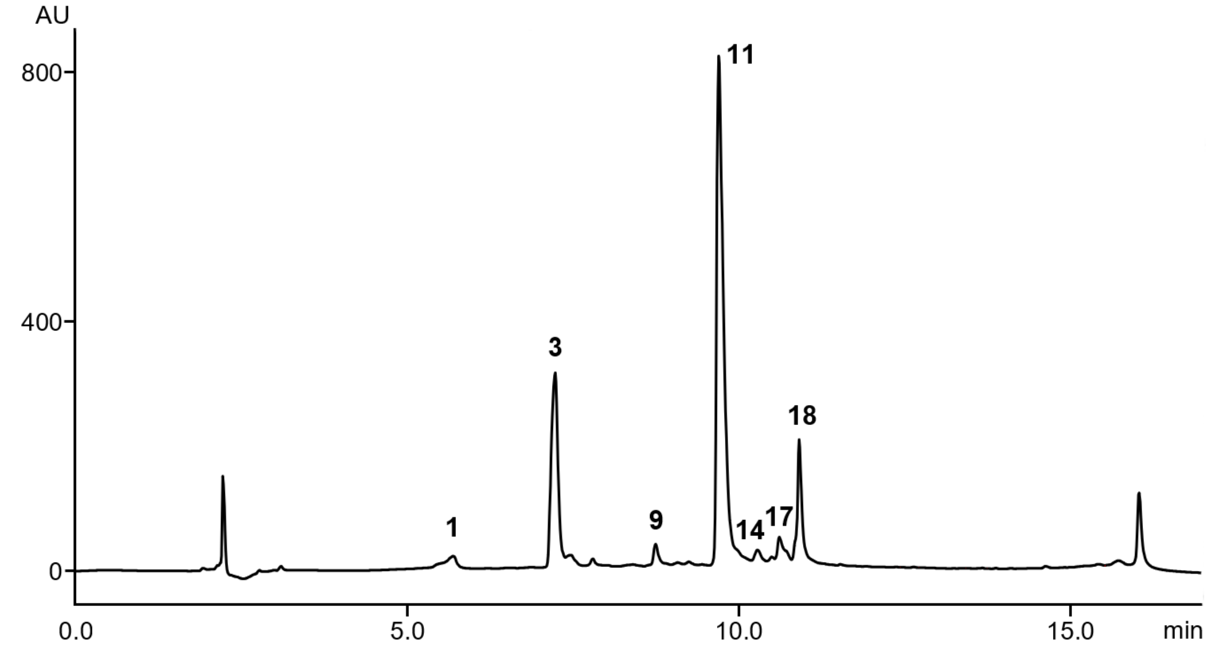

Figure S1. Typical HPLC profile of *Strongylocentrotus intermedius* total extract

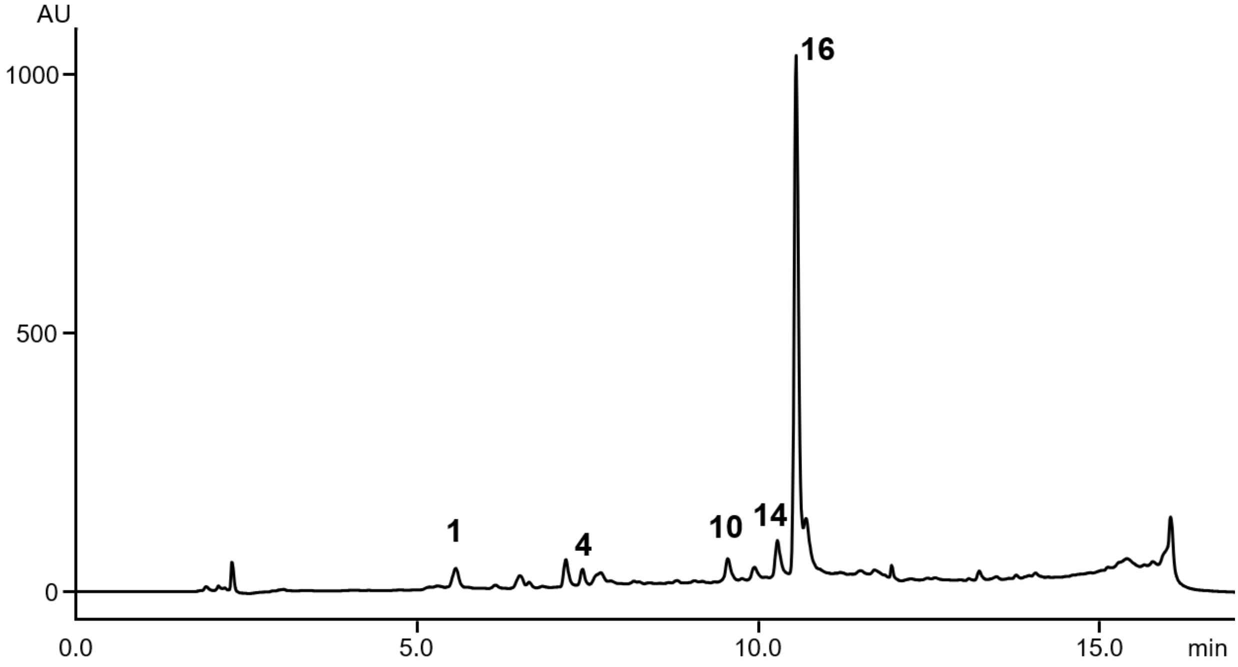

Figure S2. Typical HPLC profile of *Toxopneustes pileous* total extract

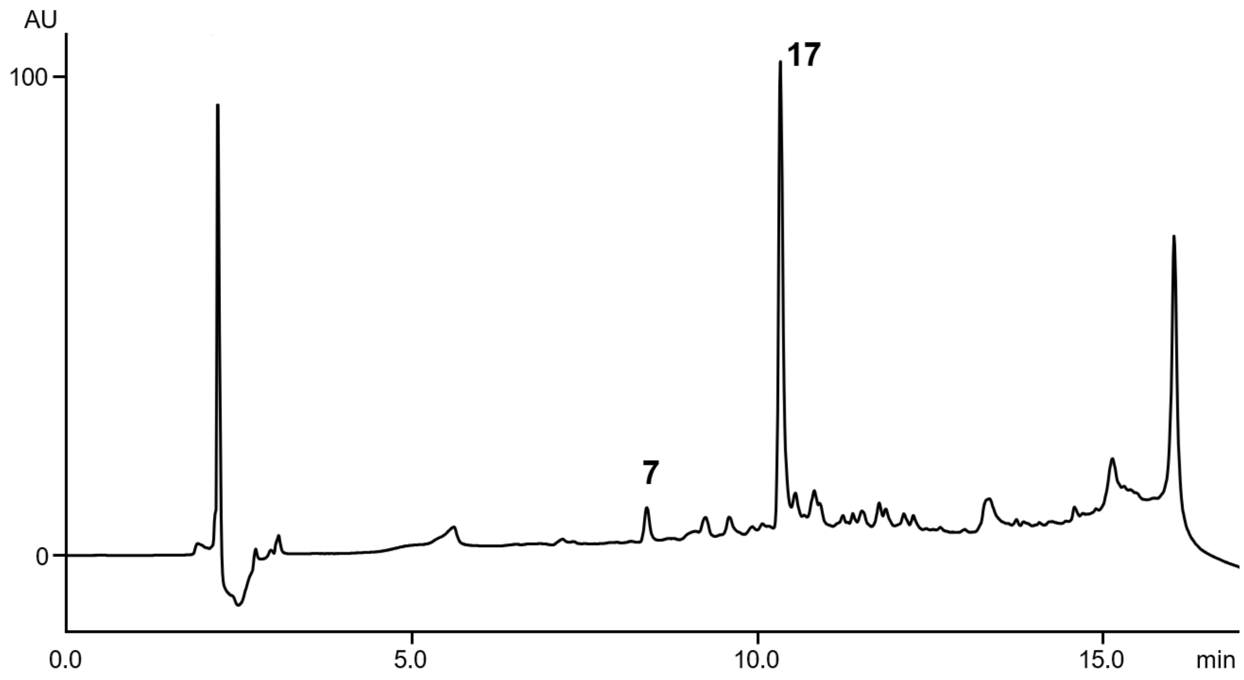

Figure S3. Typical HPLC profile of *Tripneustes gratilla* $CHCl_3$ extract

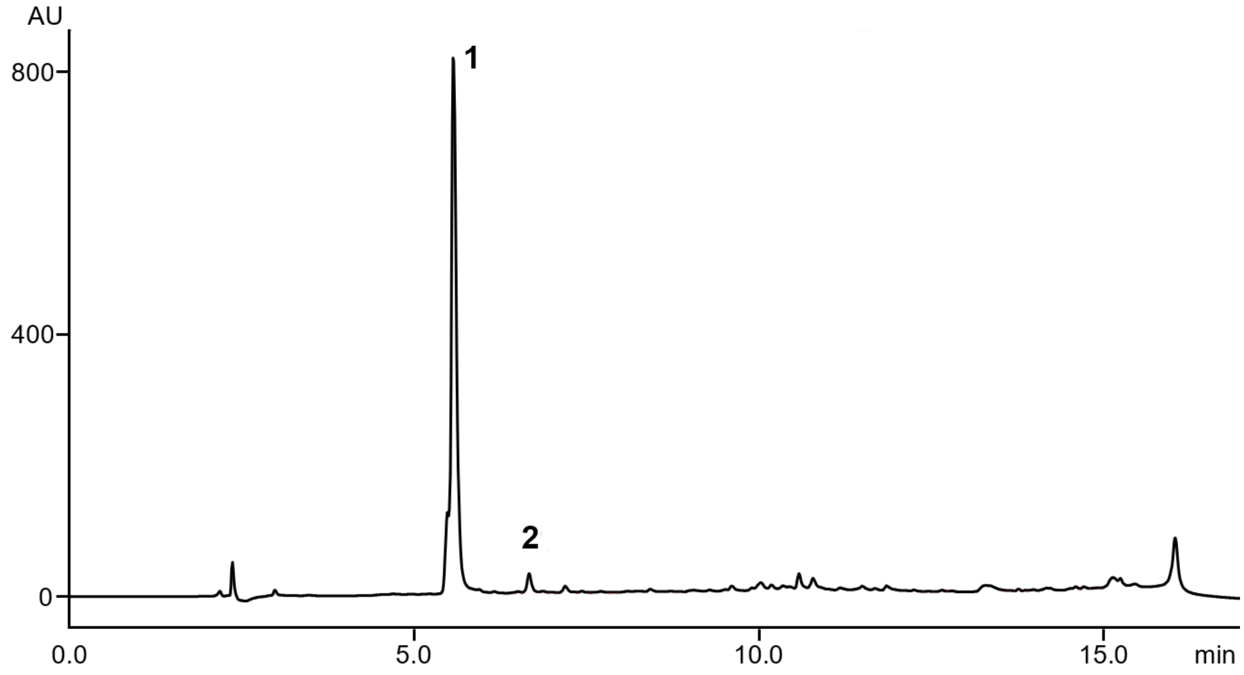

Figure S4. Typical HPLC profile of *Tripneustes gratilla* EtOAc extract

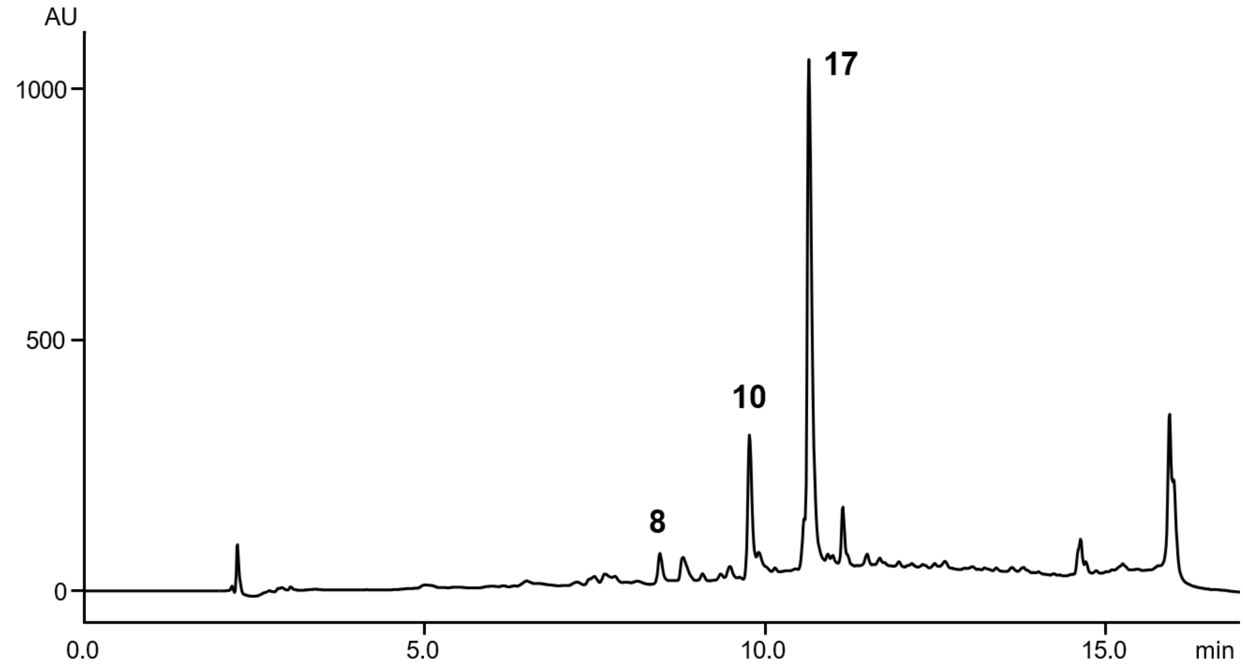

Figure S5. Typical HPLC profile of *Phyllacanthus imperialis* $CHCl_3$ extract

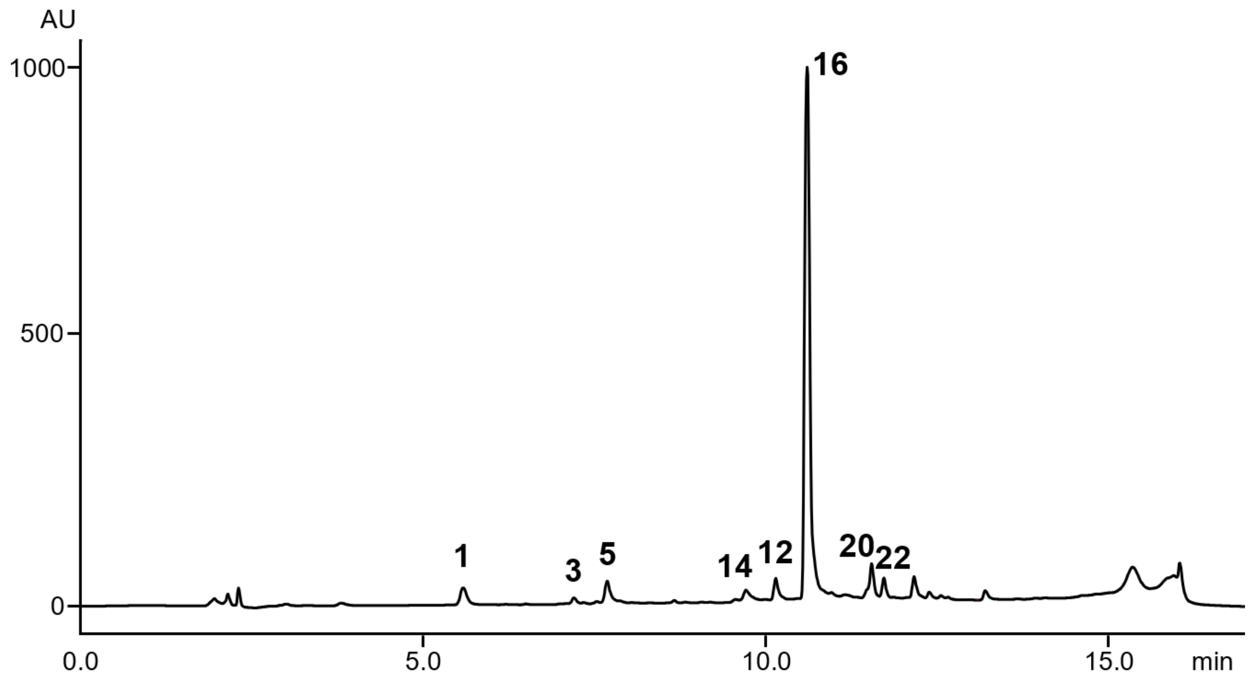

Figure S6. Typical HPLC profile of *Diadema savignyi* total extract

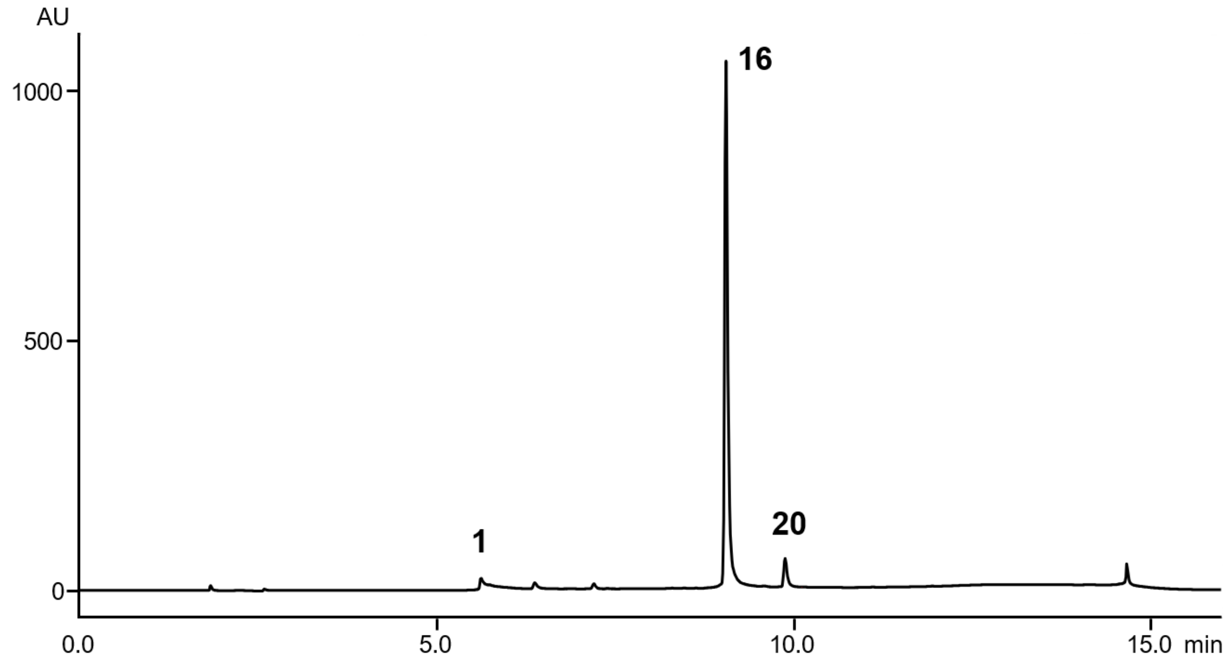

Figure S7. Typical HPLC profile of *Diadema setosum* total extract

HPLC conditions slightly differ from described in the manuscript: column Shim–pack XR-ODS (75 × 3 mm, 2,2 μm particle size, Shimadzu Europe GmbH), binary gradient of H_2O (A): MeCN (B) with the addition of 0.2% AcOH at a flow rate of 0.3 ml/min and column temp. of 40 °C. The gradient was as follows: 0 – 10 min, 20 – 100% (B); 10 – 12 min, 100% (B), 12 – 14 min, 100 – 20% (B); and 14 – 16 min, 20% (B).

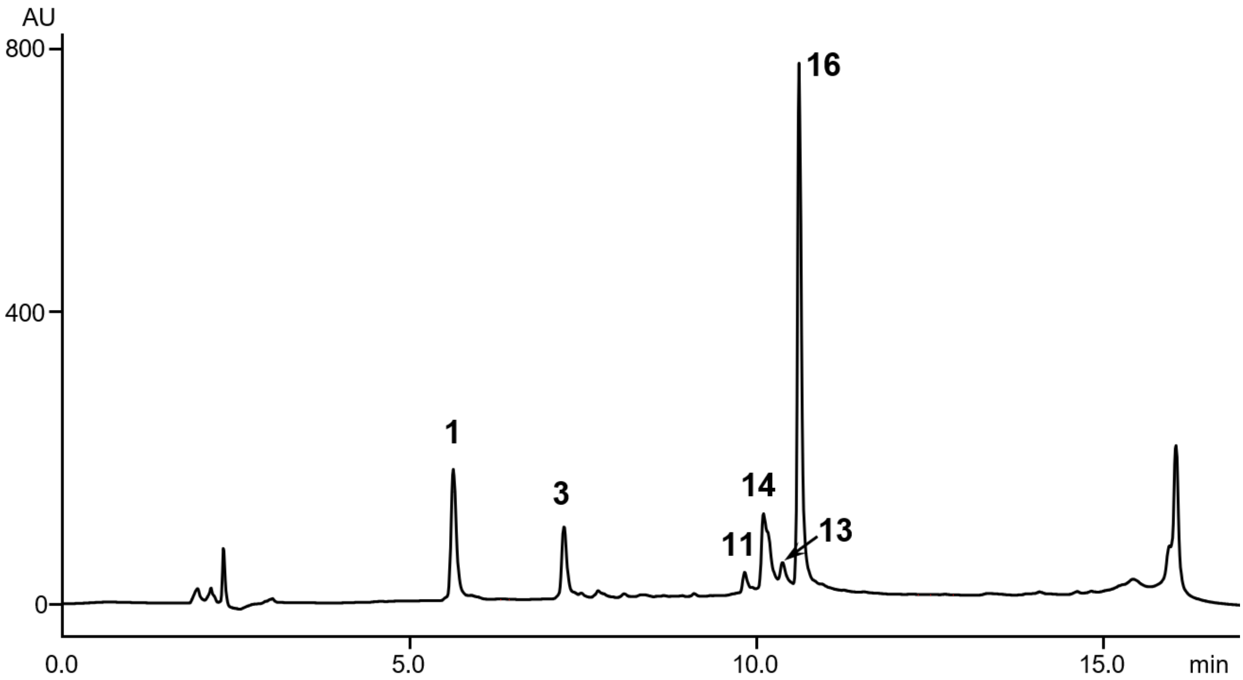

Figure S8. Typical HPLC profile of *Echinothrix calamaris* total extract

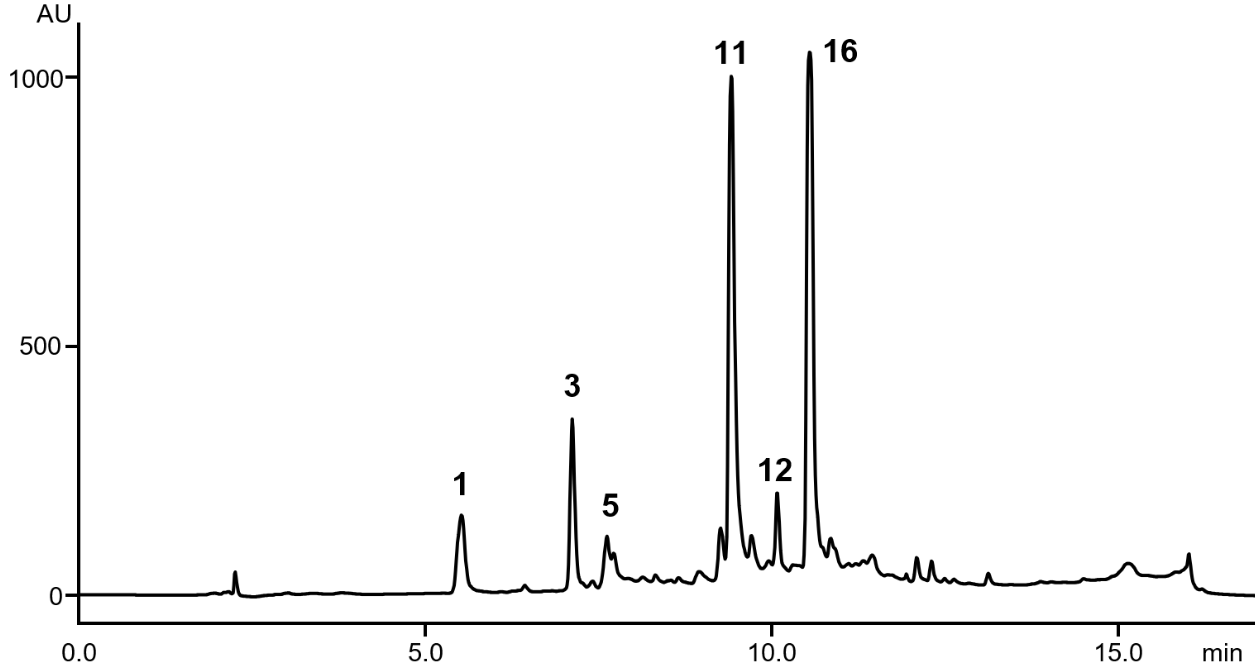

Figure S9. Typical HPLC profile of *Echinothrix diadema* total extract

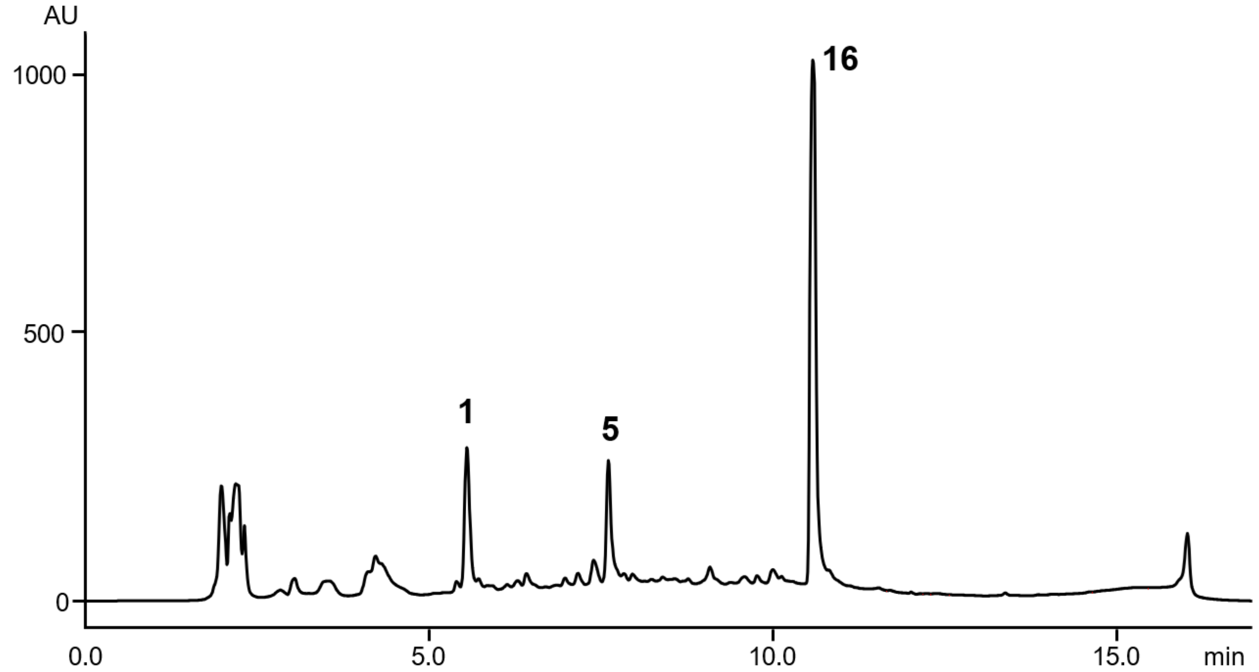

Figure S10. Typical HPLC profile of *Stomopneustes variolaris* total extract

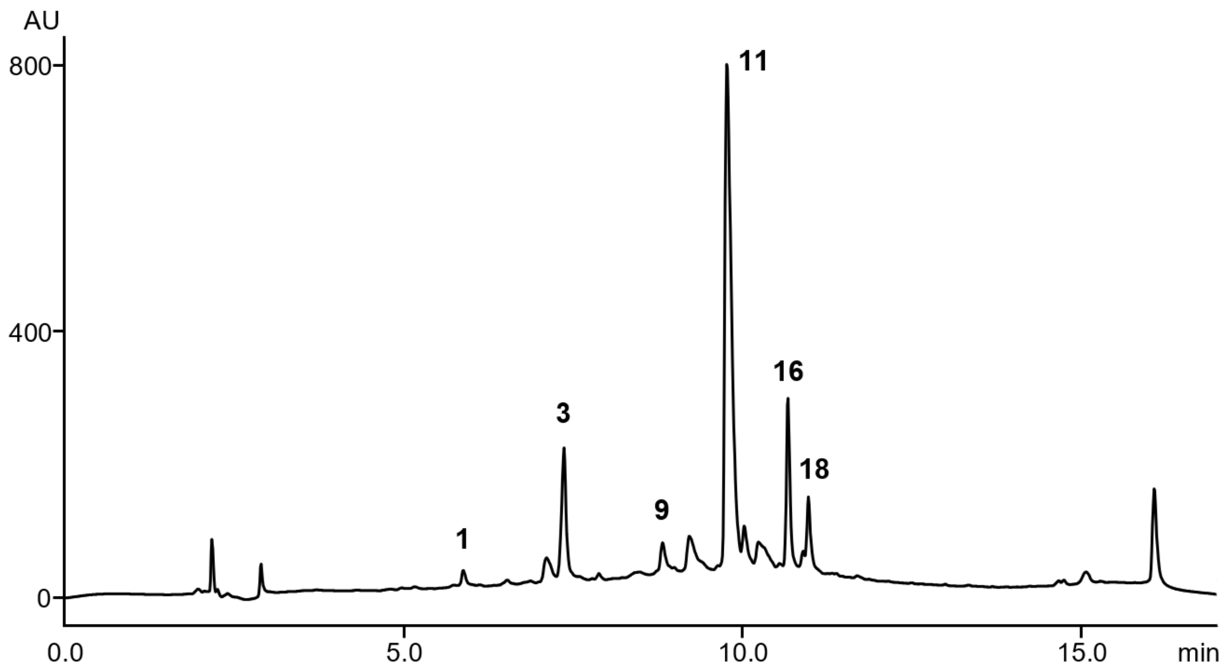

Figure S11. Typical HPLC profile of *Echinarachnius parma* total extract

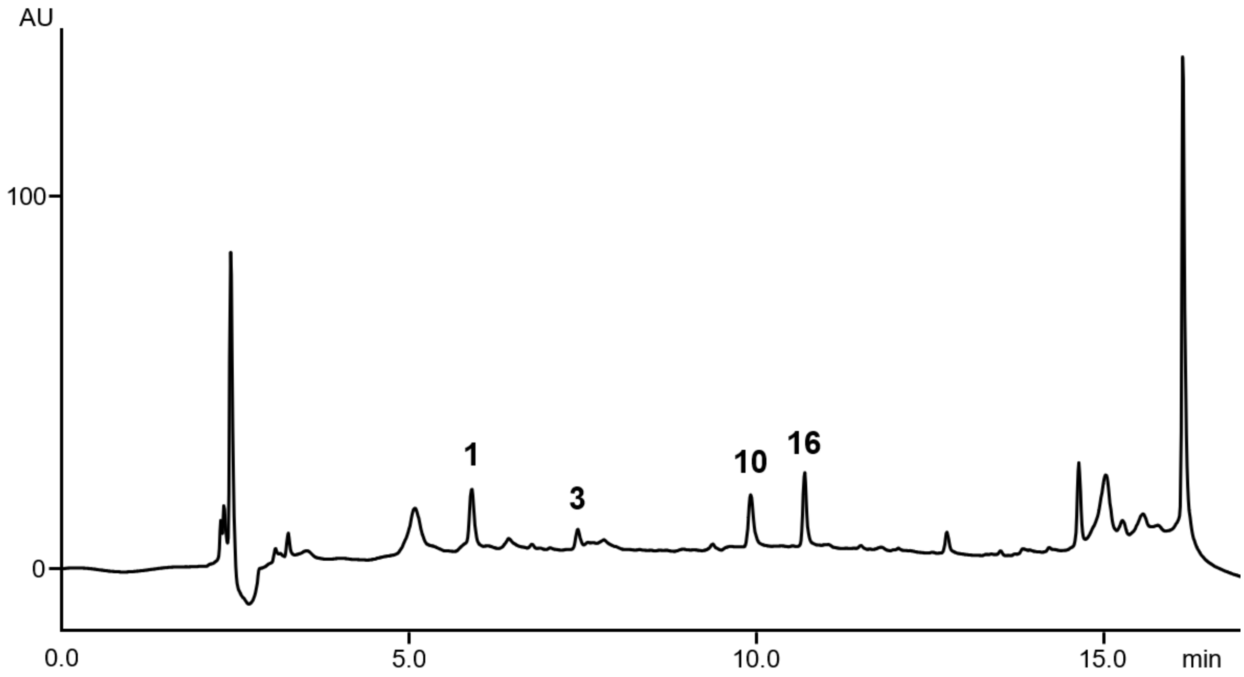

Figure S12. Typical HPLC profile of *L. decagonale* total extract

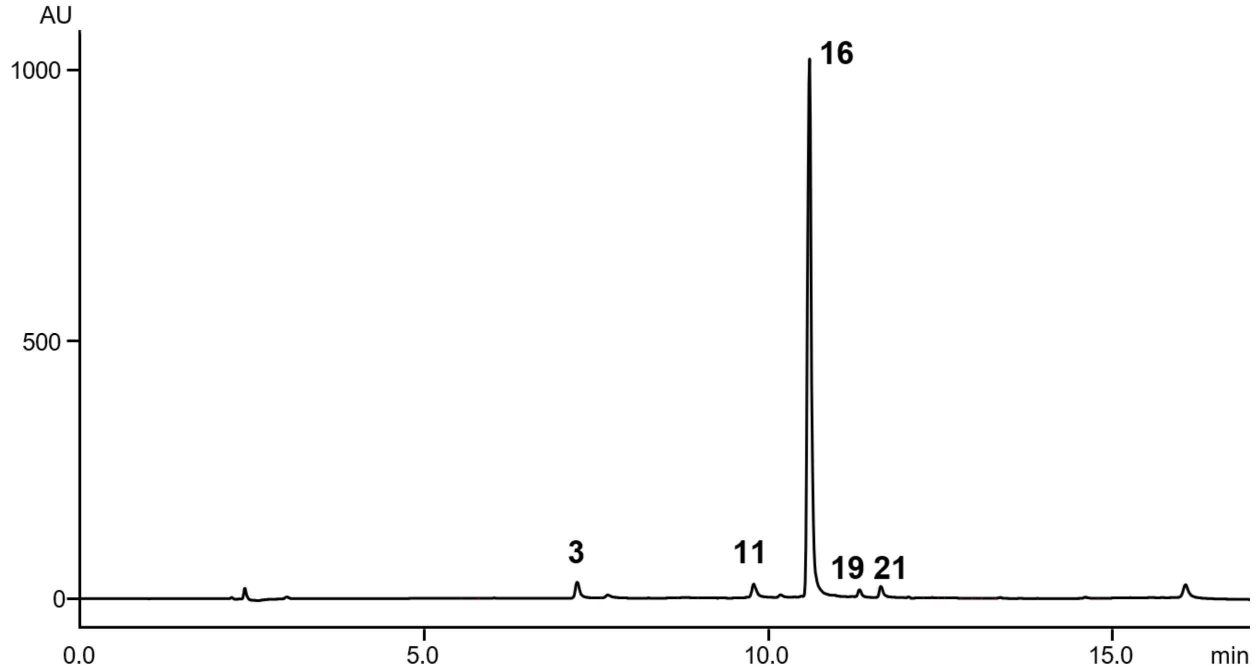

Figure S13. Typical HPLC profile of *Scaphechinus mirabilis* $CHCl_3$ extract

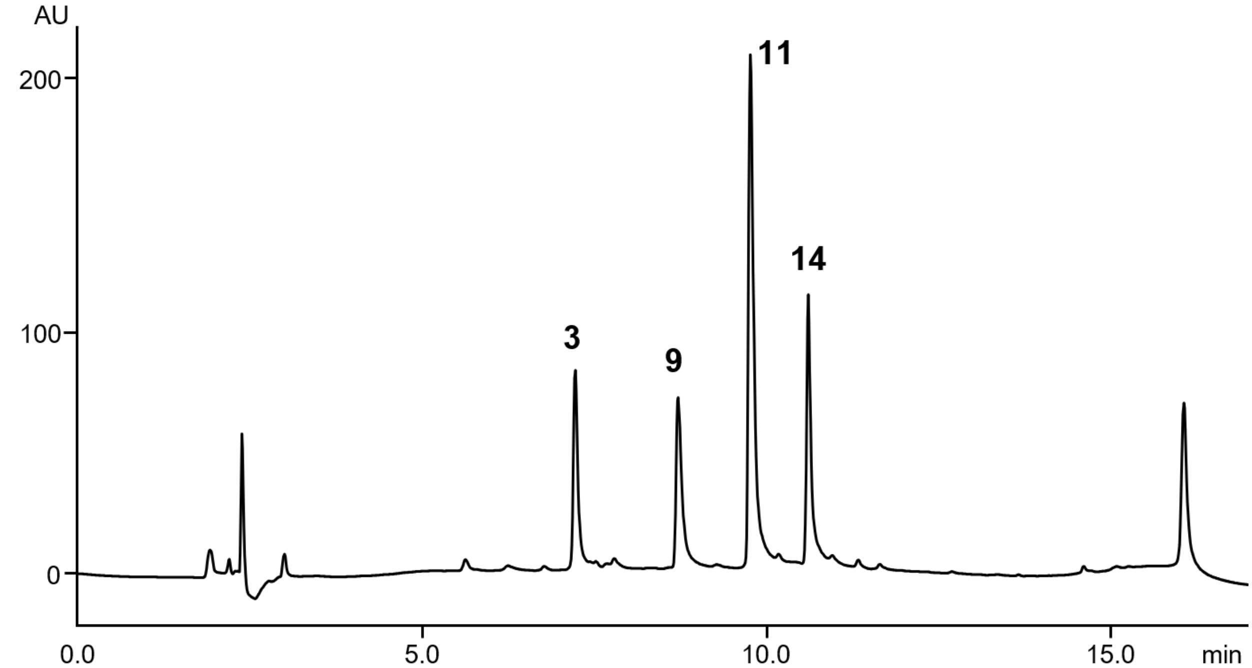

Figure S14. Typical HPLC profile of *Scaphechinus mirabilis* EtOAc extract

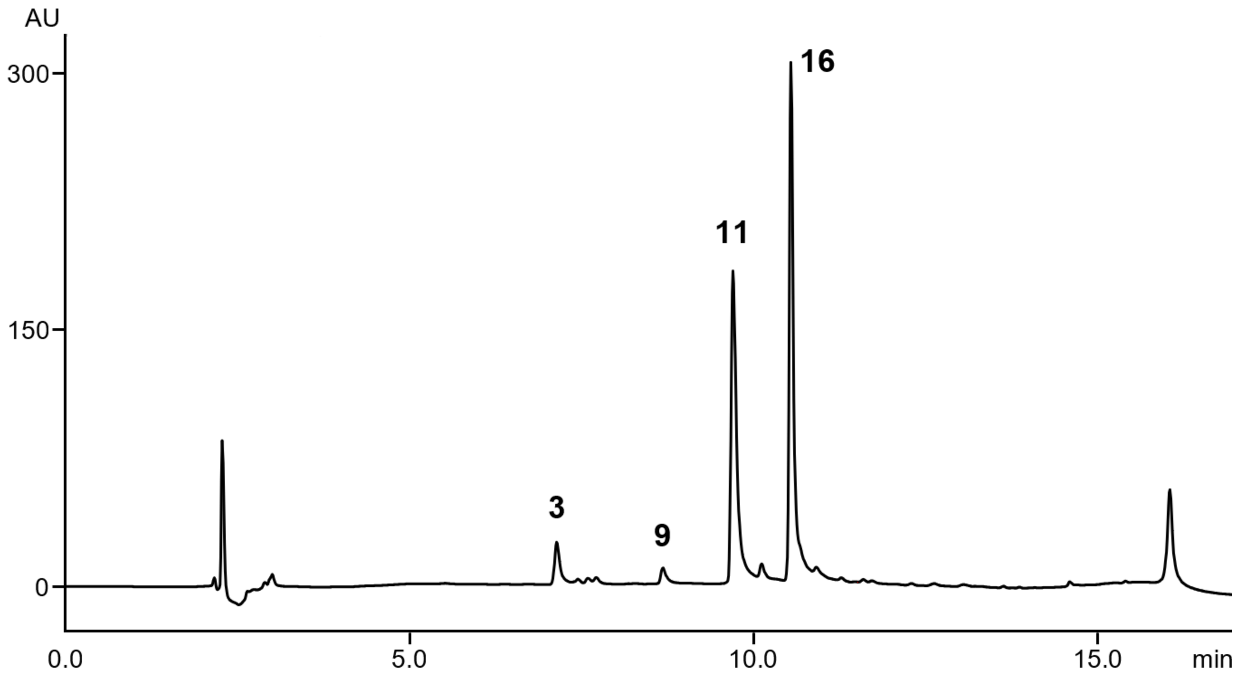

Figure S15. Typical HPLC profile of *Scaphechinus griseus* $CHCl_3$ extract

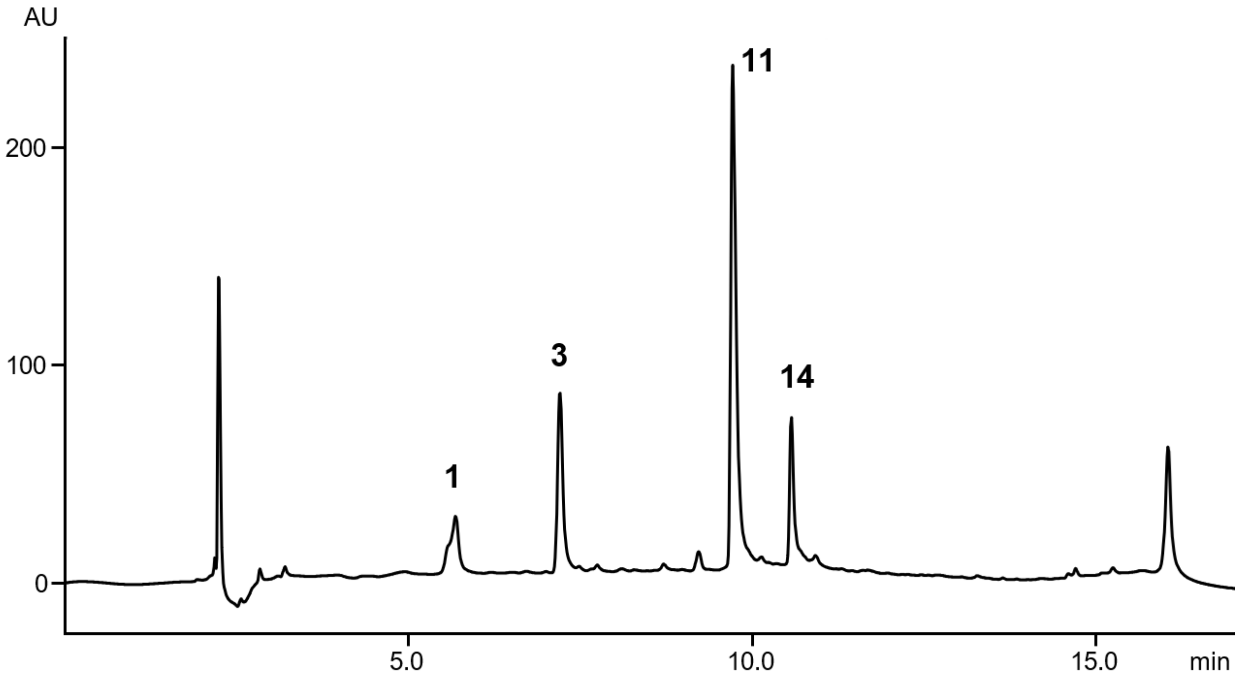

Figure S16. Typical HPLC profile of *Scaphechinus griseus* EtOAc extract

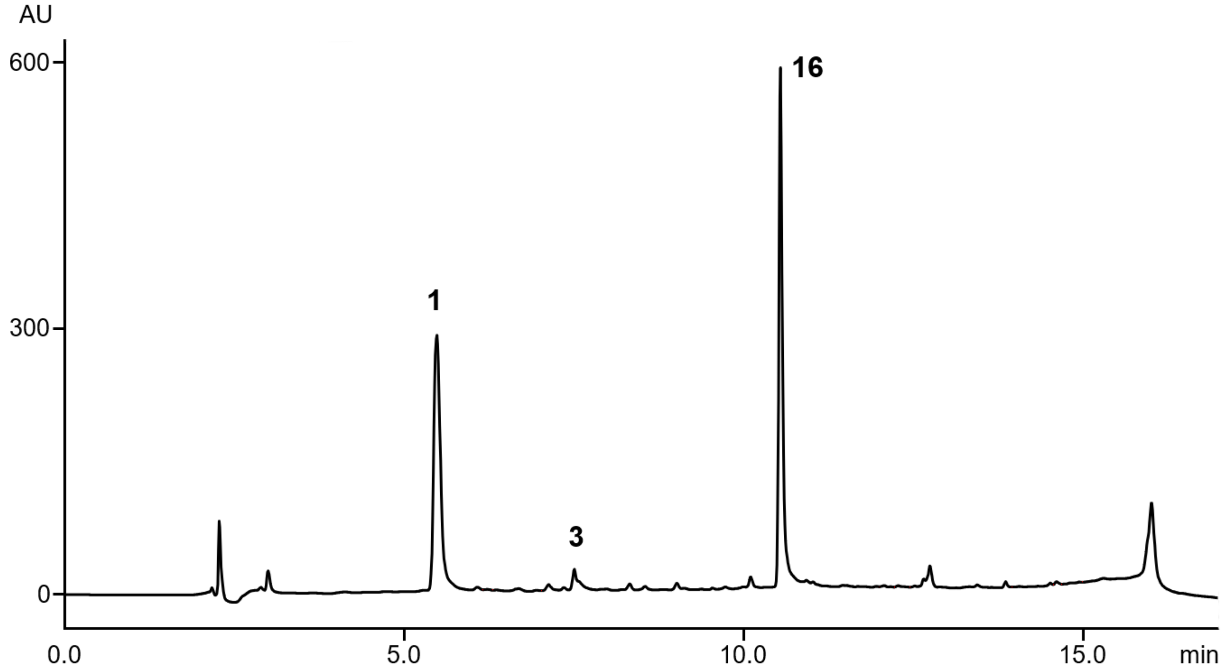

Figure S17. Typical HPLC profile of *Echinocardium cordatum* total extract

Table S2. Quinonoid pigments with established structure, found in various sea urchin species.

	Pigment name	Molecular formula	Calculated monoisotopic mass, Da	$[M–H]^-$	Absorption spectrum	Structure elucidation reference
1	Echinochrome A	$C_{12}H_{10}O_7$	266.042664	265	254, 338, 471	[1]
2	Spinochrome A	$C_{12}H_8O_7$	264.027008	263	266, 312, 508	[2]
3	Spinochrome B	$C_{10}H_6O_6$	222.016434	221	265, 320, 390, 471	[2]
4	Spinochrome C	$C_{12}H_8O_8$	280.18700	279	290, 456	[3]
5	Spinochrome D	$C_{10}H_6O_7$	238.011353	237	251, 327, 463	[1]
6	Spinochrome E	$C_{10}H_6O_8$	254.006271	253	264, 350,478	[4]
7	Echinamine A	$C_{12}H_{11}NO_6$	265.0586	264	278, 352, 477	[5]
8	Echinamine B	$C_{12}H_{11}NO_6$	265.0586	264	274, 352, 477	[5]
9	Spinamine E	$C_{10}H_7NO_7$	253.0150	252	275, 370, 473	[4]
10	Ethylidene-3,3'-bis(2,6,7-trihydroxynaphthazarin)	$C_{22}H_{14}O_{14}$	502.038361	501	254, 339, 471	[6]
11	7,7'-Anhydroethylidene-6,6'-bis(2,3,7-trihydroxynaphthazarin)	$C_{22}H_{12}O_{13}$	484.0203	483	265, 316, 470	[6]
12	Mirabiquinone	$C_{22}H_{12}O_{13}$	484.0205	483	264, 325, 452	[7]
13	7-Ethyl-3,5,6,8-tetrahydroxy-2-methoxy-1,4-naphthoquinone	$C_{13}H_{12}O_7$	280.2302	279	252, 330, 491, 525	[6]
14	7-Ethyl-2,5,6,8-tetrahydroxy-3-methoxy-1,4-naphthoquinone	$C_{13}H_{12}O_7$	280.2302	279	256, 332, 474, 497, 538	[6]
15	Namakochrome	$C_{11}H_8O_8$	268.176860	267	262, 340, 480, 524	[8]
16	Mompain	$C_{10}H_6O_6$	222.016434	221	270, 315, 515, 559	[9]
17	3-Acetyl-2-hydroxynaphthazarin	$C_{12}H_8O_6$	248.032089	247	250(sh), 296, 490, 525(sh), 568(sh)	[9]
18	Dehydroechinochrome	$C_{12}H_{12}O_9$	300.048135	299	256, 321, 391	[10]

Table S3. Quinonoid pigments with established structure that have been found at least once in sea urchins, but their nativeness has not been proven.

	Pigment name	Molecular formula	Calculated monoisotopic mass, Da	Absorption spectrum	Reference
1	6-ethyl-2-hydroxyjuglone	$C_{12}H_{10}O_4$	218.057907	237, 291, 418(sh), 432, 455(sh)	[9]
2	6-ethyl-2-hydroxynaphthazarin	$C_{12}H_{10}O_5$	234.052826	302, 479(sh), 505, 530, 544	[9]
3	3-ethyl-2,5-dihydroxybenzoquinone	$C_8H_8O_4$	168.04226	287	[9]
4	Naphthopurpurin	$C_{10}H_6O_5$	206.021530	290, 390(sh), 475, 488, 498(sh), 519(sh), 534(sh)	[9]
5	6-acetyl-2,7-dihydroxyjuglone	$C_{12}H_8O_6$	248.032089	241, 307, 374, 469	[9]
6	6-acetyl-2-hydroxynaphthazarin	$C_{12}H_8O_6$	248.032089	236, 297, 507	[9]
7	3-ethyl-2,7-dihydroxynaphthazarin (ethylmompain)	$C_{12}H_{10}O_6$	250.047745	237, 265, 321, 420(sh), 450(sh), 489(sh), 513, 549(sh)	[9], [11]
8	6-acetyl-2,3,7-trihydroxyjuglone	$C_{12}H_8O_7$	264.027008	305, 388, 490	[9]
9	6-ethyl-2,3,7-trihydroxyjuglone	$C_{12}H_{10}O_6$	250.047745	270, 330, 417, 485(sh)	[9]
10	2-hydroxy-2'-methyl-2'H-pyrano[2,3-b]naphthazarin	$C_{14}H_{10}O_6$	274.229	292, 340, 488, 564(sh)	[12]
11	3-ethyl-2,5,7,8-tetrahydroxy-1,4-naphthoquinone (ethylisomompain)	$C_{12}H_{10}O_6$	250.047745	233, 319, 423, 492, 527	[13]
12	2,3,5,8-tetrahydroxy-1,4-naphthoquinone (spinazarin)	$C_{10}H_6O_6$	222.016434	230, 246, 302(sh), 460, 489, 525(sh)	[11]
13	6-ethyl-2,3,5,8-tetrahydroxy-1,4-naphthoquinone (ethylspinazarin)	$C_{12}H_{10}O_6$	250.047745	204, 253, 292, 460, 485, 510(sh), 522(sh)	[11]
14	3-acetyl-2,7-dihydroxy-6-methyl-1,4-naphthoquinone	$C_{13}H_{10}O_7$	278.042664	261, 324, 506(sh), 535, 568(sh)	[14]

Table S4. Quinonoid pigments found in sea urchins, that have no established structure.

	**Pigment name**	**Measured [M – H]⁻**	**Molecular formula**	**Absorption spectrum**	**Detection method**	**Reference**
1	Acetylaminotrihydroxynaphthoquinone	262.0352	$C_{12}H_9NO_6$	245, 323, 484	UPLC-Q-TOFMS	[15]
2	Aminopentahydroxynaphthoquinone (Structure established by Vasileva et al. 2016, was named spinamine E)	252.0164	$C_{10}H_7NO_7$	272, 370, 484	UPLC-Q-TOFMS	[15]
3	Spinochrome dimer MW 536	535.0359	$C_{22}H_{16}O_{16}$	340, 400, 480	HPLC–DAD–MS	[16], [17]
4	Spinochrome dimer MW 484 (Authors named as anhydroethylidene-6,6′-bis(2,3,7-trihydroxynaphthazarin) 2)	483.0202	No data	No data	HPLC–DAD–MS	[16]
5	Spinochrome E sulphate derivative<br>Spinochrome E sulphate derivative iso1<br>Spinochrome E sulphate derivative iso2	332.9553<br>332.9563<br>332.9559	$C_{10}H_5O_{11}S$	260, 330, 475<br>235, 324, 502<br>No data	HPLC–DAD–MS<br>UHPLC-DAD-MS/MS<br>UHPLC-DAD-MS/MS	[18]<br>[17]<br>[17]
6	No data	255.0144	$C_{10}H_7O_8$	246, 295, 348	HPLC–DAD–MS	[18]
7	Spinochrome B sulphate derivative	300.9657	$C_{10}H_5O_9S$	262, 300, 420	HPLC–DAD–MS	[18]
8	No data	297.0249	$C_{12}H_9O_9$	220, 250, 279, 365	HPLC–DAD–MS	[18]
9	Spinochrome D sulphate derivative	316.9617	No data	231, 320, 508	UHPLC-DAD-MS/MS	[17]
10	Spinochrome dimer MW 536 sulphate derivative	614.9926	No data	257, 324, 397, 504	UHPLC-DAD-MS/MS	[17]
11	Spinochrome C sulphate derivative	358.9721	No data	225, 258, 313, 505	UHPLC-DAD-MS/MS	[17]
12	Spinochrome dimer MW 502 sulphate derivative	580.9871	No data	238, 327, 506	UHPLC-DAD-MS/MS	[17]

Table S5. Compounds found in sea urchins without proof of their quinonoid nature.

	**Pigment name**	**Measured [M – H]⁻**	**Calculated [M – H]⁻**	**Molecular formula**	**Absorption spectrum**	**Detection method**	**Reference**
1	Spinochrome D—Iso 2	237.04	237.0035	$C_{10}H_6O_7$	No data	HPLC-MS	[20]
2	Spinochrome D—Iso 3	237.04	237.0035	$C_{10}H_6O_7$	No data	HPLC-MS	[19], [20]
3	Spinochrome 252	251.02		$C_{11}H_8O_7$	No data	HPLC-MS	[20]
4	Echinamine 253<br>Most likely it is spinamine E (Vasileva et al. 2016)	252.16		$C_{10}H_7O_7N$	No data	HPLC-MS	[20]
5	Spinochrome A—Iso 2	263.01	263.01918	$C_{12}H_8O_7$	No data	HPLC-MS	[19], [20]
6	Spinochrome 282<br>Most likely it is spinochrome E dimethyl ether (Vasileva et al. 2017)	281.03	281.02975	$C_{12}H_9O_8$	No data	HPLC-MS	[20]
7	Spinochrome B—Iso 1	No data		$C_{10}H_6O_6$	No data	HPLC-MS	[21]
8	Spinochrome B—Iso 2	No data		$C_{10}H_6O_6$	No data	HPLC-MS	[21]
9	Spinochrome B—Iso 3	No data		$C_{10}H_6O_6$	No data	HPLC-MS	[21]

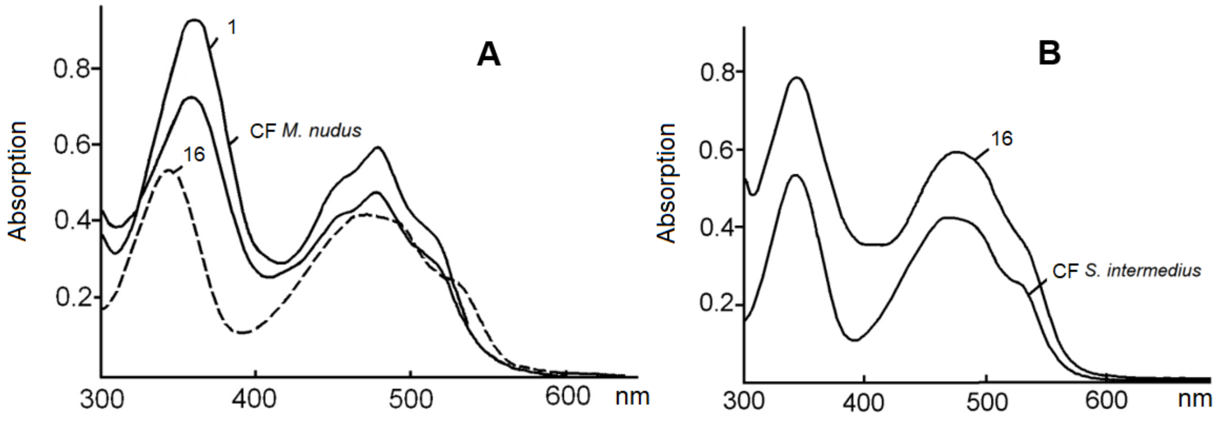

Figure S18. Absorption spectra (ethanol): **A**) echinochrome A (**16**) and spinochrome E (**1**) in comparison with the spectrum of the *M. nudus* coelomic fluid (CF) extract; **B**) echinochrome A (**16**) in comparison with the spectrum of *S. intermedius* CF.

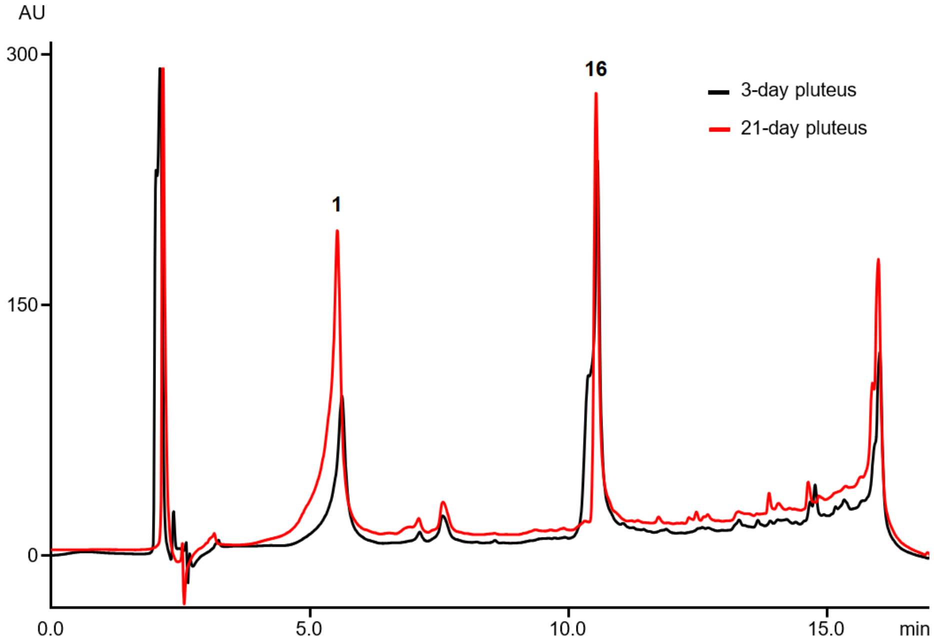

Figure S19. HPLC profile of *Scaphechinus mirabilis* 3-day and 21-day pluteus.

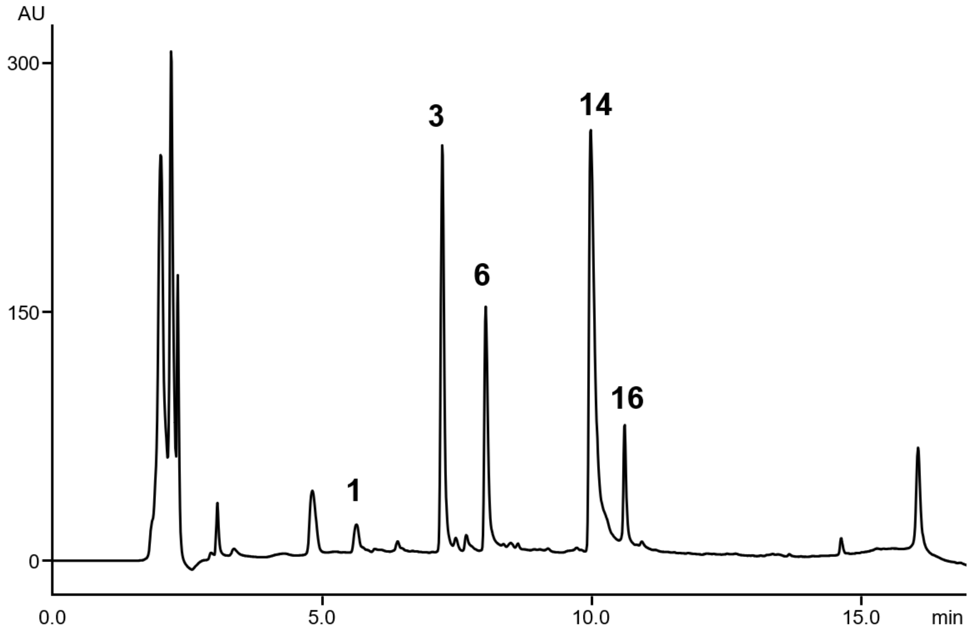

Figure S20. HPLC profile of *Echinarachnius parma* jelly-like egg membrane pigment granules

Total Quinonoid Pigments Content

The content of quinonoid pigments in sea urchin shells was determined spectrophotometrically on a UV-mini 1240 (Shimadzu Corp., Kyoto, Japan) and expressed as µg of echinochrome A equivalent per g of dry shells as described earlier. A solution of the echinochrome A standard sample was prepared at a concentration of 1.0 mg/ml in methanol. The calibration curve was prepared at five different concentration levels: 10 – 50 µg/ml, $y = 27.988x - 0.0218$, $R^2 = 0.9996$. The $CHCl_3$ and EtOAc sea urchin extracts were dissolved in methanol (20 mg/ml) and filtered through a 0.45 µm nylon syringe filter; 10 – 50 µl of the prepared solution was added to 2.5 ml of ethanol, and the absorption of each dilution at 468 nm was measured. The content of quinonoid pigments was calculated from the echinochrome A calibration curve and expressed as µg of echinochrome A equivalent per g of dry shells.

References

1. Vasileva, E.A.; Mishchenko, N.P.; Tran, V.T.T.; Vo, H.M.N.; Bui, L.M.; Denisenko, V.A.; Fedoreyev, S.A. Quinoid pigments from the sea urchin *Astropyga radiata*. *Chem. Nat. Compd*. **2017**, *53*, 356–358.
2. Thomson, R.H. *Naturally Occurring Quinones*, 2nd ed.; Academic Press: London, United Kingdom, 1971; 734 p.
3. Pokhilo, N.D.; Anufriev, V.F. Simple synthesis of spinochrome C, a sea urchin pigment. *Chem. Nat. Compd*. **2016**, *52*, 939–940.
4. Vasileva, E.A.; Mishchenko, N.P.; Zadorozhny, P.A.; Fedoreyev, S.A. New Aminonaphthoquinone from the Sea Urchins *Strongylocentrotus pallidus* and *Mesocentrotus nudus*. *Nat. Prod. Commun*. **2016**, *11*, 821–824.
5. Mischenko, N.P.; Fedoreyev, S.A.; Pokhilo, N.D.; Anufriev, V.P.; Denisenko, V.A.; Glazunov, V.P. Echinamines A and B, first aminated hydroxynaphthazarins from the sea urchin *Scaphechinus mirabilis*. *J. Nat. Prod.* **2005**, *68*, 1390–1393.
6. Vasileva, E.A.; Mishchenko, N.P.; Fedoreyev, S.A. Diversity of polyhydroxynaphthoquinone pigments in North Pacific sea urchins. *Chem.&Biodivers.* **2017**, *14*, e1700182[1–9].
7. Mishchenko, N.P.; Vasileva, E.A.; Fedoreev, S.A. Mirabiquinone, a new unsymmetrical binaphthoquinone from the sea urchin *Scaphechinus mirabilis*. *Tetrahedron Lett.* **2014**, *55*, 5967–5969.
8. Singh, H.; Moore, R.E.; Scheuer, P.J. The distribution of quinone pigments in echinoderms. *Experientia* **1967**, *23*, 624–626.
9. Moore, R.E.; Singh, H.; Scheuer, P.J. Isolation of eleven new spinochromes from echinoids of the genus *Echinothrix*. *J. Org. Chem*. **1966**, *31*, 3645–3650.
10. Novikov, V.L.; Shestak, O.P.; Mishchenko, N.P.; Fedoreev, S.A.; Vasileva, E.A.; Glazunov, V.P.; Artyukov, A.A. Oxidation of 7-ethyl-2,3,5,6,8-pentahydroxy-1,4-naphthoquinone (echinochrome A) by atmospheric oxygen 1. Structure of dehydroechinochrome. *Russ. Chem. Bull*. **2018**, 67, 282–290.
11. Yakubovskaya, A.Y.; Pokhilo, N.D.; Mishchenko, N.P.; Anufriev, V.F. Spinazarin and ethylspinazarin, pigments of the sea urchin *Scaphechinus mirabilis*. *Russ. Chem. Bull*. **2007**, *56*, 819–822.
12. Moore, R.E.; Singh, H.; Scheuer, P.J. A pyranonaphthazarin pigment from the sea urchin *Echinothrix diadema*. *Tetrahedron Lett*. **1968**, *9*, 4581-4583.
13. Mishchenko, N.P.; Fedoreev, S.A.; Glazunov, V.P.; Denisenko, V.A.; Krasovskaya, N.P.; Glebko, L.I.; Maslov, L.G.; Dmitrenok, P.S.; Bagirova, V.L. Isolation and identification of impurities in the parent substance of echinochrome and in the drug histochrome. *Pharm. Chem. J.* **2004**, *38*, 50–53.
14. Kol'tsova, E.A.; Chumak, G.N.; Maksimov, O.B. Quinoid pigments of echinodermata III. Minor pigments of the sea urchin *Strongylocentrotus nudus*. *Chem. Na.t Compd*. **1977**, *13*, 174–177.
15. Zhou, D.Y.; Qin, L.; Zhu, B.W.; Wang, X.D.; Tan, H.; Yang, J.F.; Li, D.M.; Dong, X.P.; Wu, H.T.; Sun, L.M.; Li, X.L. Extraction and antioxidant property of polyhydroxylated naphthoquinone pigments from spines of purple sea urchin *Strongylocentrotus nudus*. *Food Chem*. **2011**, *129*, 1591–1597.
16. Shikov, A.N.; Ossipov, V.I.; Martiskainen, O.; Pozharitskaya, O.N.; Ivanova, S.A.; Makarov, V.G. The offline combination of thin-layer chromatography and high-performance liquid chromatography with diode array detection and micrOTOF-Q mass spectrometry for the separation and identification of spinochromes from sea urchin (*Strongylocentrotus droebachiensis*) shells. *J. Chromatogr. A* **2011**, *1218*, 9111–9114.
17. Hira, J.; Wolfson, D.; Andersen, A.J.C.; Haug, T.; Stensvåg, K. Autofluorescence mediated red spherulocyte sorting provides insights into the source of spinochromes in sea urchins. *Sci. Rep.* **2020**, *10*, 1–9.
18. Powell, C.; Hughes, A.D.; Kelly, M.S.; Conner, S.; McDougall, G.J. Extraction and identification of antioxidant polyhydroxynaphthoquinone pigments from the sea urchin *Psammechinus miliaris*. *LWT - Food Sci. Technol.* **2014**, *59*, 455–460.
19. Brasseur, L.; Hennebert, E.; Fievez, L.; Caulier, G.; Bureau, F.; Tafforeau, L.; Flammang, P.; Gerbaux, P.; Eeckhaut, I. The roles of spinochromes in four shallow water tropical sea urchins and their potential as bioactive pharmacological agents. *Mar. Drugs* **2017**, *15*, E179 [1–18].
20. Brasseur, L.; Demeyer, M.; Decroo, C.; Caulier, G.; Flammang, P.; Gerbaux, P.; Eeckhaut, I. Identification and quantification of spinochromes in body compartments of *Echinometra mathaei*'s coloured types. *R. Soc. Open Sci.* **2018**, *5*, 171213 [1–15].
21. Brasseur, L.; Caulier, G.; Lepoint, G.; Gerbaux, P.; Eeckhaut, I. *Echinometra mathaei* and its ectocommensal shrimps: the role of sea urchin spinochrome pigments in the symbiotic association. *Sci. Rep.* **2018**, *8*, 1–10.
